# Metabolism of glioblastoma: a review of metabolic adaptations and metabolic therapeutic interventions

**DOI:** 10.3389/fonc.2025.1712576

**Published:** 2025-12-10

**Authors:** James Chung, Jawad Saad, Ahmad Kafri, Julien Rossignol, Maxwell Verbrugge, Jesse Bakke

**Affiliations:** 1 Department of Foundational Sciences, College of Medicine, Central Michigan University, Mt. Pleasant, MI, United States; 2 Program in Neurosciences, Central Michigan University, Mt. Pleasant, MI, United States; 3 Internal Medicine Residency, School of Medicine, Indiana University, Indianapolis, IN, United States

**Keywords:** glioblastoma, metabolism, cancer, cancer signaling, brain cancer, metabolic therapeutics

## Abstract

Glioblastoma (GBM) is the most common and aggressive primary malignancy of the central nervous system, marked by profound metabolic reprogramming that promotes growth, invasion, and therapeutic resistance. This review examines metabolic adaptations that sustain GBM progression and summarizes current and emerging strategies that target these pathways. GBM cells display increased aerobic glycolysis, glutaminolysis, lipid and cholesterol synthesis, and mitochondrial remodeling. These processes are regulated by oncogenic alterations such as EGFR amplification, PTEN loss, and HIF-1α stabilization, which allow tumor cells to thrive in hypoxic and nutrient-poor environments. Accumulation of lactate further supports metabolic flexibility and promotes an immunosuppressive microenvironment. Recent studies have focused on exploiting these metabolic vulnerabilities through dietary, pharmacologic, and oxygen-modulating interventions. The ketogenic diet has been explored as an adjuvant therapy to reduce glucose availability and enhance treatment sensitivity. Pharmacologic approaches include inhibition of key metabolic enzymes such as hexokinase 2, pyruvate kinase M2, pyruvate dehydrogenase kinase, and glutaminase. Additional strategies aim to disrupt mitochondrial function through VDAC1 blockade or to reduce tumor hypoxia using hypoxia-activated prodrugs, hyperbaric oxygen therapy, and oxygen-transporting agents. Preclinical findings suggest these approaches can suppress tumor proliferation and improve responsiveness to radiation and chemotherapy, although clinical evidence remains limited. Combining metabolic interventions with standard therapies may help overcome GBM’s intrinsic resistance and metabolic plasticity. Overall, the review highlights metabolism as a key determinant of GBM pathophysiology and a promising target for therapeutic innovation, emphasizing the importance of continued translational research to identify and exploit context-specific metabolic vulnerabilities in this highly lethal disease.

## 1 Introduction

Glioblastoma (GBM) is the most common primary malignancy of the central nervous system and is associated with an exceedingly poor prognosis. GBM accounts for 49% of all malignant primary brain and central nervous system (CNS) tumors in adult patients (1), with approximately 13,000 cases diagnosed in the United States each year (2). It is a high-grade subtype of glioma, a grouping which also includes astrocytomas, oligodendrogliomas, and ependymomas. Among these, GBM is the most frequent and aggressive entity, with incidence increasing with age and peaking in older adults. In population-based studies and clinical trials, the five-year survival rate is approximately 5-10% for patients who receive standard-of-care treatment (3, 4), including maximally-safe surgical resection followed by radiotherapy with concurrent and adjuvant temozolomide (5, 6). Unfortunately, despite modern therapeutic approaches, GBM is still considered a terminal diagnosis with median survival of 14 months (7). Survival beyond five years is exceedingly rare, with a 10-year survival rate of less than 1% (8). Even new research examining novel approaches such as tumor-treating fields, immunotherapies, and targeted agents has yet to show a substantial improvement in the long-term clinical course of GBM (9, 10).

The primary challenges posed in the treatment of GBM are multifactorial and rooted deeply in the tumor’s pathophysiology and clinical behavior. Resistance to chemotherapy and radiotherapy is both intrinsic and acquired: the blood-brain barrier makes delivery of chemotherapeutic drugs to neoplastic cells difficult, and GBM itself is molecularly heterogeneous and often features robust DNA repair mechanisms such as enhanced methylated-DNA-protein-cysteine methyltransferase (MGMT) activity (11). Metabolic reprogramming by neoplastic cells is a central driver of resistance to therapy, and directly contributes to the challenges of treatment. The combination of upregulated aerobic glycolysis (Warburg effect), enhanced glutamine/lipid metabolism, and rerouting of metabolic flux enables tumor cells to thrive in hypoxic, nutrition-depleted environments (12, 13). Several pharmacologic interventions aimed at targeting key glycolytic enzymes have been investigated, including drugs such as dichloroacetate (an inhibitor of pyruvate dehydrogenase), shikonin (an inhibitor of pyruvate kinase), and others discussed further in this paper. While some success with glycolytic inhibitors has been demonstrated in preclinical studies, GBM cells can become resistant and escape via metabolic plasticity and use of alternative substrates such as amino acids, lipids, or glycogen (14–16). Combination strategies that target multiple metabolic pathways simultaneously are currently under investigation (17). However, in the current clinical landscape, recurrence of disease is inevitable, regardless of the therapy regimen. Essentially all patients experience tumor progression due to the highly infiltrative nature of GBM; complete surgical removal is practically impossible, and rapid regrowth unavoidably occurs from residual malignant cells.

This review examines the metabolic landscape of GBM, with a focus on the widespread reprogramming of energy pathways that enables tumor cells to adapt and thrive in harsh microenvironments. Core pathways including glycolysis, glutaminolysis, and mitochondrial function are highlighted for their effects on tumor growth, therapy resistance, and immune evasion. Key genetic and molecular alterations and how they contribute to oncogenesis are discussed, such as mutations in isocitrate dehydrogenase (IDH), phosphatase and TENsin homolog (PTEN) loss with downstream PI3K/AKT/mTOR activation, and epidermal growth factor receptor (EGFR) amplification. Building on this mechanistic foundation, emerging metabolic therapies designed to exploit GBM’s vulnerabilities are reviewed, ranging from dietary interventions such as the ketogenic diet to pharmacologic inhibitors targeting steps in cellular metabolism and hypoxia signaling. Collectively, this review explores the emerging potential of integrating metabolic strategies into existing treatment regimens in the pursuit of improved outcomes for patients with this otherwise devastating malignancy.

## 2 The metabolic landscape of glioblastoma

### 2.1 Key mutations driving metabolic reprogramming

**Figure 1:**
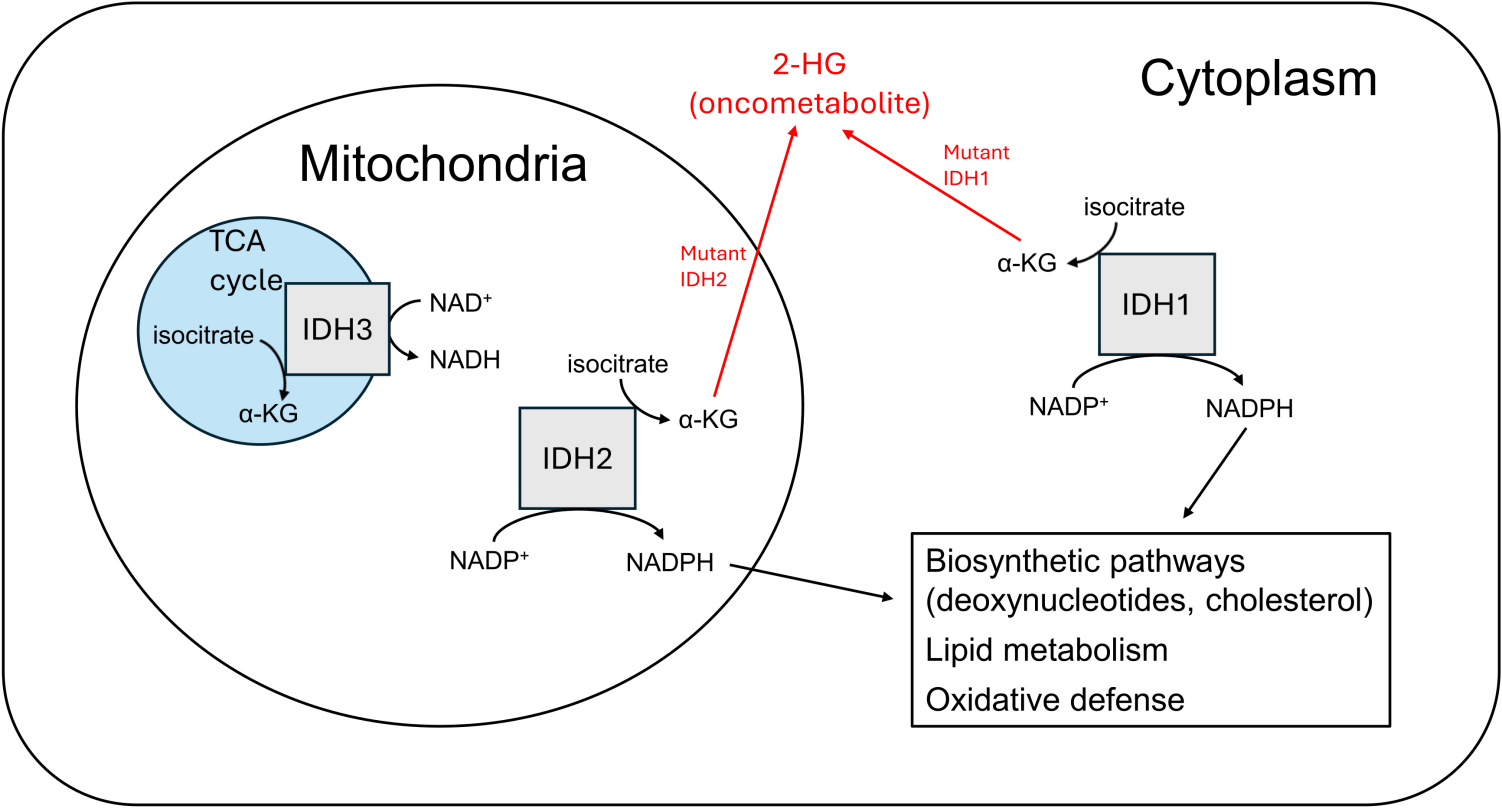
IDH isoforms differ by cofactor use, localization, and metabolic function.

Among the multitude of genetic and molecular aberrations that have been identified in GBM, several have been implicated as key determinants in the metabolic reprogramming that contributes to the pathogenesis and progression of this devastating disease. The presence of mutated or wild-type IDH has become a key element of the current World Health Organization (WHO) 2021 classification system of CNS tumors (18). Three isoforms of the IDH enzyme are present in humans: IDH1, IDH2, and IDH3, each having unique cellular localization and metabolic functions (19–22) (**Figure 1**). Mutations in IDH1 (primarily) and IDH2 have been detected in up to 70% of WHO grade II and III gliomas, and are common in secondary GBMs that can arise from these lower grade malignancies (23). Genomic analyses have revealed specific somatic mutations at codon 132 of the IDH1 gene in a higher proportion of secondary GBMs (85%) as compared to primary lesions (5%) (24). These data, alongside *in vitro* research showing mutant IDH produces the onco-metabolite D-2-hydroxyglutarate (2-HG) that can promote tumorigenic phenotypes (25, 26), suggests that this may be a significant factor in the secondary progression of lower grade gliomas (e.g. astrocytomas and oligodendrogliomas) to GBM rather than the generation of primary tumors. 2-HG has been demonstrated to inhibit histone demethylation, specifically the Jumonji family histone lysine demethylase KDM4C, thereby impairing the expression of genes important in normal cellular differentiation (27). There are other possible mechanisms by which this phenomenon is reinforced as well: in hypoxic conditions, such as the local environment of a rapidly growing tumor, the activity of lactate dehydrogenase A (LDHA) is upregulated to support increased glycolytic rate by regenerating NAD^+^ in the reduction of pyruvate to lactate. However, LDHA has also been shown to metabolize alpha-ketoglutarate (α-KG) to the L-(S)-enantiomeric form of 2-HG (L-2-HG), with similar inhibitory effects on histone demethylation (28).

IDH-mutant gliomas tend to have a missense mutation at codon 132 of IDH1, most commonly R132H, or at codons R140 or R172 of IDH2, such as R140Q or R172K. These mutations replace an arginine residue with histidine, glutamine, or lysine. This allows for an abnormal enzymatic conversion of alpha-ketoglutarate (α-KG) to 2-HG. It is thought that 2-HG may serve as an oncometabolite by impairing cellular functioning via a variety of epigenetic and metabolic mechanisms (29). Notably, patients who have a genetic deficiency of the enzyme 2-HG dehydrogenase are unable to convert 2-HG back to α-KG, and subsequently develop accumulations of the metabolite in the brain; these patients have been shown to be at increased risk of leukoencephalopathy and brain tumors (30, 31). CNS tissue has an exceptional ability to take up glutamate via excitatory amino acid transporters (EAATs), leading to a high level of substrate readily available to be converted to α-KG (and subsequently 2-HG, with its potential for downstream tumorigenic effects.) This may explain why IDH1 mutations are highly prevalent in many CNS malignancies like GBM and could play an important role in tumor progression. Additionally, mutated IDH1 isoforms are impaired in their ability to synthesize nicotinamide adenine dinucleotide phosphate (NADPH) and may in fact consume it in the reduction of α-KG to 2-HG (32). The cellular depletion of this molecule could potentially contribute to malignancy via increased susceptibility to DNA mutations (33), as NADPH plays an important role in protection against oxidative damage via antioxidant substrates like glutathione. However, the role of disrupted NADPH production in the pathophysiology of GBM remains unclear and is not well-characterized.

2-HG is known to interfere with glutaminergic signaling and the functioning of α-KG dependent enzymes, including those that are central to the regulation of hypoxia-inducible factor 1-alpha (HIF1α) and vascular endothelial growth factor (VEGF) (34). Physiologically, HIF1α and VEGF are upregulated in response to tissue hypoxia to promote neo-angiogenesis. High levels of 2-HG have been shown to increase cellular levels of HIF1α and VEGF even in the absence of hypoxia, a phenomenon which has been termed “pseudohypoxia” (35, 36). HIF-1α plays a direct role in the preferential shift in cellular metabolism to aerobic glycolysis, a process known as the Warburg effect. This is a well-known phenomenon in tumor biology and has been identified as a crucial step in the pathogenesis of GBM that contributes to its aggressive nature (37). It is important to note however that the vast majority of GBM have wild-type IDH1 and still have dysregulation of HIF-1α and VEGF, indicating that this key process occurs most commonly via mechanisms other than those involving the presence of 2-HG. Amplification of EGFR signaling has been observed in 35-45% of GBM with wild-type IDH (38), most commonly occurring via an exon deletion that leads to a constitutively active receptor (39). This leads to activation of the PI3K/Akt/mTOR pathway independent of ligand binding, causing downstream increases in cellular anabolism and inhibition of apoptosis, as well as neo-angiogenesis via increased levels of HIF-1α and VEGF (40, 41). Dysregulation of this same pathway has also been shown to occur via mutations, deletions, or suppression of PTEN (20-40% of IDH wild-type GBM). PTEN is a tumor suppressor and phosphatase that negatively regulates phosphoinositide 3-kinases (PI3K), and its loss similarly leads to increased HIF-1α and VEGF (42). Experimental data have demonstrated that co-occurrence of EGFR amplification and PTEN loss may be synergistic and predispose to chromosomal instability and an aggressive GBM phenotype (43).

The categorization of GBM into molecular subtypes has evolved over the years. Most recently in 2017, Wang et al. (44) built upon previous work by Phillips et al. (45) and Verhaak et al. (46) by proposing the following classification: IDH mutant and IDH wild-type, with further subclassification of the wild-type form into proneuronal (PN), classical (CL), and mesenchymal (MES) (47). While IDH wild-type tumors do not produce 2-HG, they do demonstrate some subtype-specific features that indirectly influence tumor metabolism. For example, the PN subtype often features platelet-derived growth factor receptor alpha (PDGFRA) amplification, which is linked to enhanced glycolysis (48, 49); the CL subtype is driven by the aforementioned EGFR activation that promotes glycolytic flux and growth signaling; and the MES subtype is notable for neurofibromin 1 (NF1) loss and nuclear factor kappa-light-chain-enhancer of activated B cells (NF-κB) activation, processes associated with metabolic reprogramming under inflammatory and immune pressure (50, 51).

### 2.2 Aerobic glycolysis and the Warburg effect

One of the hallmarks of GBM is the metabolic reprogramming that allows GBM tumor cells to proliferate and adapt in heterogeneous environments. Due to constantly changing levels of vascularity throughout a tumor microenvironment (TME) and differing levels of oxygen and nutrient availability, having the ability to be metabolically flexible is crucial to GBM resilience and tumorigenesis. GBM cells, like other cancer cells, demonstrate the renown “Warburg effect”. The Warburg effect is the preference for cancer cells to metabolize glucose by lactic acid fermentation to generate adenosine triphosphate (ATP) despite being in the presence of oxygen, a process called aerobic glycolysis. This is in contrast to how normal cells metabolize glucose to generate ATP in the presence of oxygen, via oxidative phosphorylation and the citric acid cycle (52), a process called respiration. Understanding the metabolic advantages that GBM cells, and cancer cells in general, gain by utilizing both respiration and aerobic glycolysis begins with understanding the trade-off between efficiency versus speed. The speed of glycolysis compensates for its inefficiency: in the time it takes a normal cell to metabolize one glucose molecule into 36 ATP via respiration, a cancer cell can process 10 glucose molecules into 20 lactic acid molecules, generating 20 ATP through glycolysis (52). Thus, in normoxic conditions a cancer cell can process 11 glucose molecules to generate 56 ATP. Under anoxic conditions, cancer cells may convert 13 glucose molecules into 26 ATP, still maintaining competitiveness. These rapid cycles result in cancer cells producing 10–13% more ATP overall than normal cells, despite being less efficient on a per-glucose basis. More important than energy production, this accelerated process generates precursor metabolites for tumor cells to proliferate. Glycolysis allows GBM cells to divert glycolytic intermediates toward biosynthetic pathways such as the pentose phosphate pathway (PPP), nucleotide synthesis, and amino acid production, supporting anabolic growth and redox balance without compromising energy supply. These insights are reflected in recent TME physiologic MRI studies showing that approximately two-thirds of vital GBM tumor tissue is dominated by aerobic glycolysis, with a glycolysis-to-OxPhos ratio of 38% to 19%, while a significant 24% of the tumor also displays hypoxia (53). These findings emphasize the metabolic heterogeneity of GBM and highlight glycolysis not just as an energy strategy, but as a fundamental driver of tumor proliferation and biosynthesis.

GBM cells maintain their energy supply through these processes while simultaneously solving the problem of growth. Producing more biomass and constructing new cancer cells requires the ability to generate more biosynthetic metabolites, such as DNA, RNA, proteins, and structural membrane lipids (54, 55). Additionally, pyruvate, the end product of glycolysis, may be metabolized into acetyl-CoA via the mitochondria and exported as citrate, which in turn fuels fatty acid and cholesterol synthesis—both upregulated in GBM (55).

Besides providing the GBM cell with anabolic metabolites, glycolysis generates lactate, which equips the cancer cell with certain crucial metabolic advantages. Lactate is responsible for the flexibility and adaptability of GBM cells in different TMEs within the same tumor. The high levels of lactate generated by glycolysis enable GBM cells to smoothly interconvert between aerobic glycolysis and oxidative phosphorylation (56, 57). Lactate does this by serving as a signal factor inducing the expression of proteins and transporters in the local environment, mainly monocarboxylate transporter 1 (MCT1) and monocarboxylate transporter 4 (MCT4). MCT1 is a H+/lactate symporter that takes up lactic acid while MCT4 is a H+/lactate symporter that effluxes lactic acid. It has been found in GBM that glucose transporter type 1 (GLUT1), HIF-1α, lactate dehydrogenase (LDH), and MCT4 were significantly expressed in the interior region of the tumor, whereas MCT1, C-MYC, and nuclear respiratory factor 1 (NRF1) were significantly expressed in the lateral region (57). These findings show that interior regions of GBMs, generally regions with decreased vascularity, take up glucose and produce ATP via aerobic glycolysis. The interior region then generates increasing amounts of lactate from its high level of glycolysis, as the level of lactate grows so does the strength of the signal it has on the expression of HIF-1α and local MCT symporters. The lactate leads to acidification of the environment and stabilization of activated HIF-1α in the interior region which then reinforces glycolysis in the interior region by upregulating GLUT1, LDHA, and hexokinase 2 (HK2), key proteins involved in the glycolytic pathway. The lactate levels increase the expression of MCT4 in the interior region leading to higher levels of lactate being effluxed to the lateral region of the GBM where the increased expression of MCT1 allows those cells to take up the lactate being effluxed to it. Once allocated to the lateral regions of GBM, the lactate is used for oxidative phosphorylation and generation of ATP, aided by the enhanced levels of C-MYC (an OXPHOS regulatory protein) and NRF1, a transcription factor that increases the activity of oxidative phosphorylation (OXPHOS). This unique ability to transfer lactate across different regions within a tumor allows GBM to be metabolically flexible and utilize both ATP pathways to proliferate in the face of different conditions.

**Figure 2:**
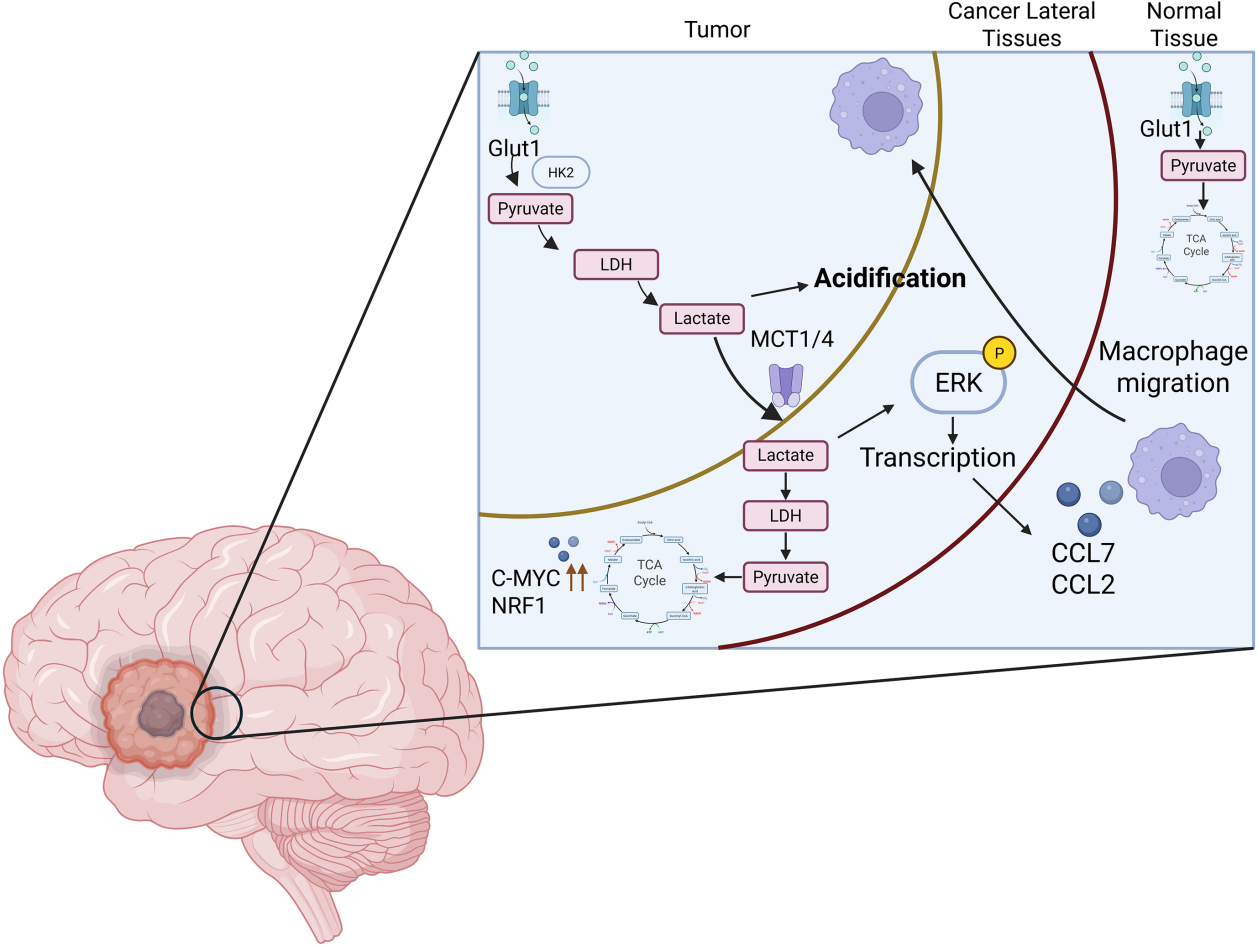
Interplay between aerobic glycolysis, lactate shuttling, and immune modulation in GBM.

The unique abundance of lactate in the GBM TME also provides neoplastic advantages by influencing the local immune phenotype leading to immune resistance (**Figure 2**). GBM is considered an immunologically cold tumor with a very low burden of T-cells in the TME responsible for its poor response to conventional immunotherapy. However, the immune phenotype of GBM is much more nuanced than simply lacking T-cells, the TME is composed of a robust infiltration of macrophages and microglial cells. Microglial cells already present in the local GBM environment in addition to the recruited macrophages from the arterial periphery comprise 30% to as high as 70% of infiltrating cells in the TME (56, 58). There is complex interplay between lactate and these immune cells. Recent studies have uncovered that LDHA-derived lactate modulates the GBM TME by triggering the ERK signaling cascade. This cascade leads to increased expression of the chemokines CCL2 and CCL7, enhancing recruitment of tumor-associated macrophages (TAMs) into the TME. These infiltrating macrophages not only suppress anti-tumor immunity but also reinforce tumor growth by secreting LDHA-enriched extracellular vesicles that further promote glioma cell glycolysis and proliferation (59). Lactate continues to have profound effects on the local macrophages and microglial cells, driving them to adopt different functional states based on signaling in their environment. There is the classically activated M1 macrophage state associated with pro-inflammatory effects, anti-tumor signals, stimulation of cytotoxic T-cells, secretion of cytokines like interleukin (IL)-12, tumor necrosis factor alpha (TNF-α), reactive oxygen species (ROS) and essentially activity to kill tumor cells and pathogens (60). Then there is the M2 macrophage state associated with anti-inflammatory effects, tissue repair and remodeling, promotion of angiogenesis, wound healing, suppression of T-cell responses, secretion of cytokines like IL-10 and transforming growth factor beta (TGF-β), and expression of arginase 1 (ARG1), VEGF, and CD206 among others, essentially activity to promote tumor progression (56, 58). The lactate accumulation from aerobic glycolysis acidifies the TME and promotes the M2 tumor-associated macrophage (TAM) polarization of these macrophages and microglial cells. Lactate enhances the HIF-1α stabilization in macrophages, which upregulates ARG1 and VEGF expression—hallmarks of the M2 phenotype. Lactate also acts on these immune cells epigenetically by increasing H3K9 acetylation in macrophages at genes like ARG1 and Retnla, reinforcing M2 gene expression and locking TAMs into an M2 polarized state (61). It has been shown that low CD74/high M2 signature is linked to increased tumor aggressiveness, while CD74 expression, associated with M1 macrophages, correlates with longer patient survival (62).

Lactate’s immune influence does not stop with tumor-associated macrophage function, it extends to T-cells as well. It has been shown that lactate directly influences Regulatory T-cells (Tregs) (56, 63). The physiological role of Treg cells is to maintain immune tolerance by suppressing effector T-cell activity and preventing inappropriate immune responses. This function is critical for preserving self-tolerance and preventing autoimmunity. However, when Treg cells become pathologic, they contribute greatly to tumor immune evasion. Studies show that lactate increases ubiquitin-specific peptidase 39 (USP39) expression in Treg cells, a crucial part of the RNA splicing complex, leading to USP39-dependent RNA-splicing mediated cytotoxic T-lymphocyte-associated protein 4 (CTLA-4) expression in a forkhead box P3 (foxp3)-dependent manner (63). In other words, high levels of lactate in the GBM TME modulates RNA splicing only in the Treg cell (Foxp3-dependent, meaning it does not affect RNA splicing in other T-cells) to increase CTLA-4 expression on the Treg cell surface, thus enhancing its immunosuppressive effects and preventing effector T-cells from infiltrating and attacking the GBM tumor cells. CTLA-4 is an important immune checkpoint receptor expressed on T-cells that downregulates immune responses and maintains T-cell self-tolerance. CTLA-4 functions by outcompeting the co-stimulatory receptor CD28 for binding to ligands CD80 and CD86 on antigen-presenting cells, leading to an inhibitory signal that dampens T-cell activation (64).​ Essentially, the glycolytic lactate in GBM promotes Treg cell function and tumor evasion of the body’s immune system.

Lactate also serves a role in the TME to directly affect T effector lymphocytes such as CD8+ T-cells (56, 65). Short-term exposure to tumor-derived lactic acid rapidly impairs CD8+ cytotoxic T lymphocytes by reducing their proliferation, suppressing cytokine production (IL-2 and interferon gamma (IFN-γ)), and decreasing cytolytic ability through the loss of perforin and granzyme B. This immunosuppressive effect is driven not just by acidic pH in the TME, but by a combined lactate–proton mechanism mediated through MCT1 transporters (65). Because activated CD8+ T-cells depend on sustained glycolysis, excess extracellular lactate disrupts lactate efflux, leading to intracellular acidification and metabolic dysfunction that blunt T-cell activity within the GBM TME. The MCT1 transporters on the CD8+ T-cells are unable to operate efficiently due to the disrupted gradient of lactate, thereby leading to metabolic dysfunction inside of the T-cell.

### 2.3 Fatty acid synthesis

GBM cells are capable of altering lipid metabolism and increasing the synthesis of fatty acids through several coordinated mechanisms. Key enzymes such as fatty acid synthase (FASN), ATP-citrate lyase (ACLY), and elongases such as ELOVL6, are activated transcriptionally via oncogenic signaling and epigenetic modifications (66). Upregulation of these pathways serves to support *de novo* lipogenesis. Additionally, activity of acetyl-CoA carboxylase (ACC) is increased; one of the two isoforms of this enzyme, ACC1, catalyzes the carboxylation of acetyl-CoA to malonyl-CoA which is the rate-limiting step in fatty acid biosynthesis. Some studies have demonstrated that inhibition of ACC1/ACC2 in GBM cells reduces proliferation, indicating that activity of these enzymes may be necessary to support GBM growth (67). Interestingly however, clinical data has shown that lower ACC1 expression is associated with poor survival rates, which may suggest that there is a context-dependent tumor suppressor role for this enzyme in certain populations (68). More recent evidence demonstrates that although reduced ACC1 activity may decrease fatty acid synthesis, there is a paradoxical promotion of a pro-tumorigenic phenotype due to increased availability of acetyl-CoA for use by the enzyme histone acetyltransferase P300. This leads to upregulation of DNA methyltransferase 1, resulting in hypermethylation and suppression of the succinate dehydrogenase (SDH) gene. Decreased SDH activity elevates levels of ROS species and promotes migration and invasion of GBM cells (68).

Similarly to fatty acids, cholesterol synthesis is often dysregulated (and upregulated) in GBM, supporting tumor growth and survival. Unlike normal astrocytes, which suppress cholesterol production under conditions such as high cell density, GBM cells frequently have sustained activation of the cholesterol biosynthetic pathway (69). This persistent activity is driven by loss of cell cycle control through defects in pathways such as p53 and retinoblastoma (RB) genes. Ultimately, there is continuous stimulation of the mevalonate pathway, leading to increased cholesterol production and accumulation within tumor cells (68, 70). Sterol regulatory element-binding protein 2 (SREBP2), the master transcriptional regulator of cholesterol biosynthesis, is highly active in GBM. It drives the expression of key enzymes, including HMG-CoA reductase (HMGCR) and lanosterol synthase, and also regulates genes such as LDLR which codes for the low-density lipoprotein receptor. This activity is associated with enhanced proliferation and migration of GBM cells (71, 72).

### 2.4 Glutaminolysis

**Figure 3:**
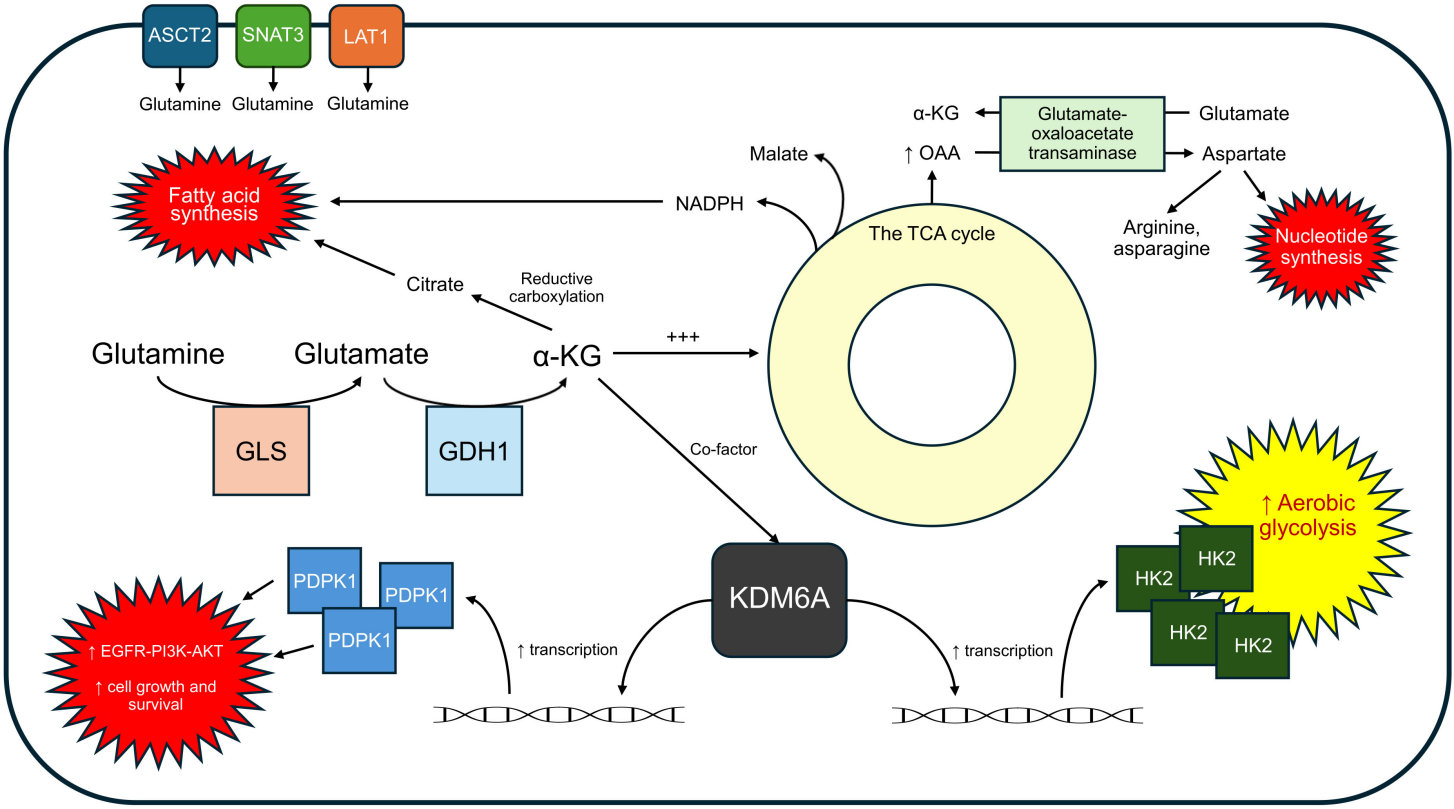
Glutaminolysis supports GBM growth through NADPH production, anaplerosis, and signal amplification.

Glutaminolysis plays a pivotal role in GBM metabolism by supporting fatty acid synthesis by producing a flux of NADPH (a reduced cofactor) via malic enzyme activity, so much so that it appears to provide an abundance of NADPH for other anabolic processes such as nucleotide production on top of primarily lipid synthesis (68). The glutaminolytic process can be summarized as follows: glutamine is converted into α-KG which enters the tricarboxylic acid (TCA) cycle and is ultimately converted into malate. The conversion of malate into pyruvate via malic enzyme generates this robust NADPH production. It is important to understand that glycolysis is still the primary source for carbon in fatty acid production in GBM. However, glutamine does provide a good portion of carbon for fatty acid production on top of its primary role of NADPH generation, as much as 25% of total fatty acyl carbon (73). Glutamine derived carbons also result in aspartate, a major precursor for the production of nucleotides, arginine, and asparagine. Another major role of glutamine metabolism is the provision of oxaloacetate (OAA) in order to sustain the TCA cycle, a process called anaplerosis. Glutaminolysis serves as a major anaplerotic pathway in GBM, replenishing OAA in the TCA cycle to compensate for the loss of intermediates like citrate siphoned for lipid synthesis. While acetyl-CoA is primarily derived from glucose, OAA is predominantly supplied by glutamine, making glutaminolysis essential for sustaining mitochondrial metabolism, supporting the continuous generation of biosynthetic precursors, and fueling rapid tumor growth (**Figure 3**).

In GBM, the role of glutaminolysis depends not only on how glutamine is used but also on the enzymes and transporters that help bring it into the cell and break it down. The enzyme glutaminase (GLS) converts glutamine into glutamate, allowing it to enter the TCA cycle and support other important growth pathways. Glutamine transporters like ASCT2 (SLC1A5), SNAT3 (SLC38A3), and LAT1 (SLC7A5) are often increased in GBM cells to keep a steady supply of glutamine coming in. Blocking GLS or these transporters has been shown to reduce tumor metabolism and growth, making them promising targets for treatment (74).

Another key enzyme in the glutaminolysis pathway is glutamate dehydrogenase 1 (GDH1), which catalyzes the conversion of glutamate to α-KG. In GBM, this reaction has effects beyond metabolism: α-KG acts as a cofactor for KDM6A, a histone demethylase that removes H3K27me3, a repressive epigenetic molecule. This demethylation specifically occurs at the promoter region of phosphoinositide-dependent protein kinase-1 (PDPK1), increasing its transcription. Elevated levels of PDPK1 then amplify the EGFR–PI3K–AKT signaling pathway, which is known to support GBM cell growth and survival (68). This shows that GDH1 does not just help fuel the cell, it also helps turn on key growth signals in GBM by linking metabolism to gene expression.

Beyond its role in signal amplification, GDH1-catalyzed glutaminolysis also contributes to the metabolic reprogramming of GBM cells by promoting glycolysis (13). This occurs through the upregulation of HK2 in a process dependent on KDM6A-mediated demethylation of the HK2 promoter. This glycolysis-promoting effect of GDH1 occurs even under high-glucose conditions, highlighting its importance not just as a backup to glucose metabolism, but as a central driver of GBM metabolic activity (75). The activity of HK2 has been shown to play a key role in the progression of malignant tumors, with increased expression associated with poorer prognosis in GBM and various other cancers. Concordantly, loss of HK2 *in vivo* leads to decreased vascular proliferation and increased radiosensitivity (76). While glutaminolysis and glycolysis are typically thought of as parallel nutrient pathways, here we see glutamine metabolism actively enhances glucose metabolism, reinforcing the tumor’s metabolic flexibility and aggressiveness.

### 2.5 HIF-1α and hypoxia in GBM metabolism

GBM is characterized by significant intratumoral hypoxia resulting from rapid proliferation and abnormal vasculature. To survive and adapt to these low-oxygen conditions, GBM cells rely on the transcription factor HIF-1α, which becomes stabilized under hypoxic stress. Once stabilized, HIF-1α translocates to the nucleus and binds hypoxia response elements (HREs) in the promoter regions of key metabolic genes. This transcriptional activity directly upregulates GLUT1, HK2, and LDHA, promoting aerobic glycolysis and reinforcing the Warburg effect (77). This metabolic shift enables GBM cells to maintain ATP production, generate biosynthetic intermediates, and manage redox balance even in oxygen-poor environments. Interestingly, HIF-1α activity in GBM is not limited to classic hypoxic responses, it also remains active in normoxic settings. GBM cells have evolved mechanisms to stabilize HIF-1α under normoxic conditions, thus, amplifying their metabolic adaptability. Oncogenic signaling pathways such as PI3K/AKT/mTOR and RAS/RAF/MEK/ERK, play a central role in this process (78). These pathways enhance HIF-1α protein translation and inhibit its degradation, allowing HIF-1α to remain active in oxygen-rich tumor regions. Mammalian target of rapamycin (mTOR), in particular, promotes cap-dependent translation of HIF-1α mRNA (79), while PI3K/AKT signaling dampens prolyl hydroxylase activity, reducing HIF-1α hydroxylation and preventing ubiquitin-mediated proteasomal degradation (80). The result is sustained transcriptional activity by HIF-1α. This constitutive expression underscores HIF-1α’s pivotal role in maintaining the unique metabolism of GBM, regardless of oxygen availability.

**Figure 4:**
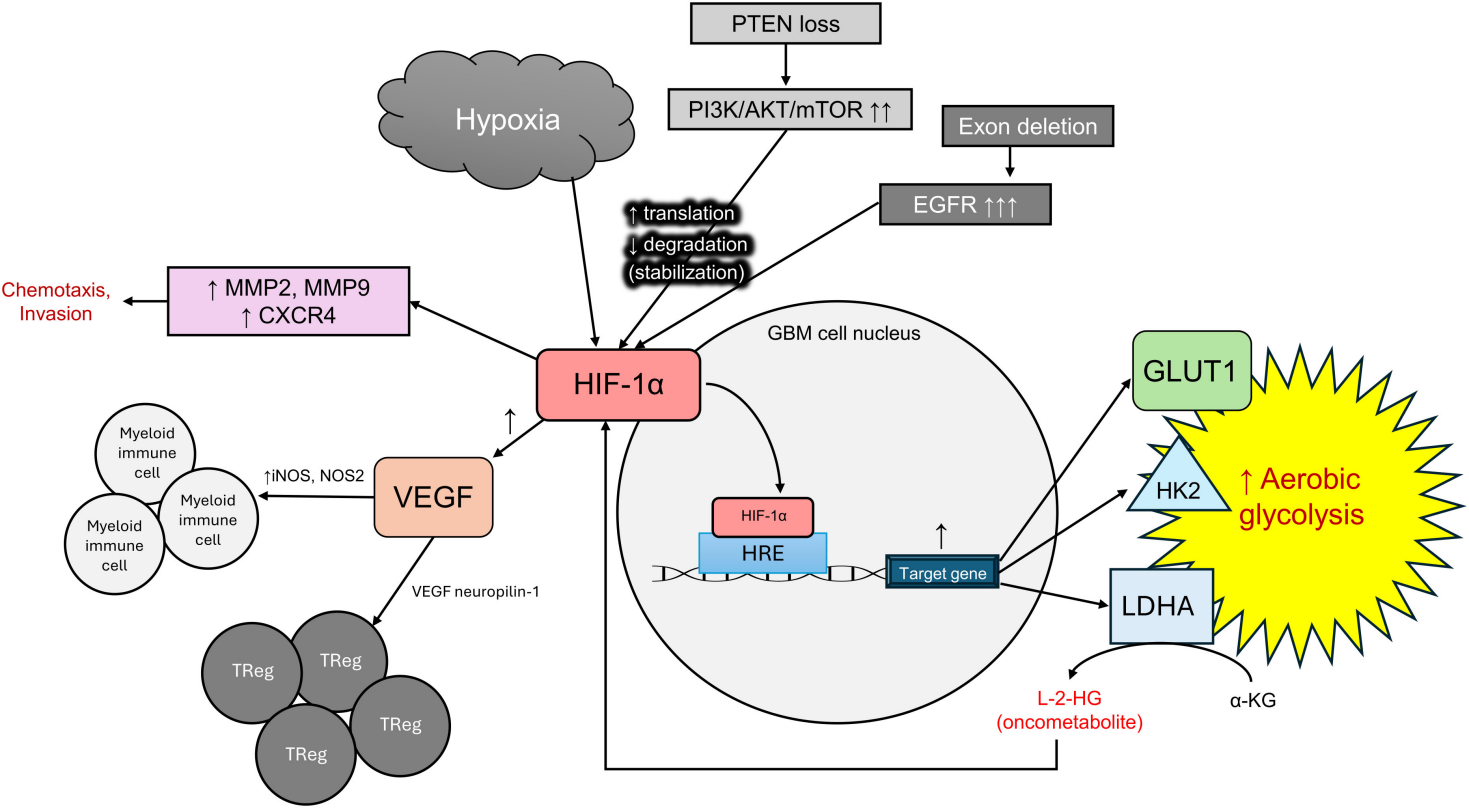
HIF-1α as a regulator of metabolic reprogramming, invasion, and immune modulation in GBM.

HIF-1α plays many roles in GBM progression, not only contributing to metabolic reprogramming but also fueling invasion and immune modulation of GBM cells. In the hypoxic TME, stabilization of HIF-1α not only enhances glycolytic flux through the upregulation of HK2 and PDPK1, but also supports tumor cell motility and invasiveness (77). This is achieved in part through the transcriptional activation of genes such as MMP2 and MMP9, which encode matrix metalloproteinases that degrade extracellular matrix barriers (81), as well as CXCR4, a chemokine receptor important in glioma cell migration along stromal cell-derived factor 1 (SDF-1) gradients. GBM cells expressing CXCR4 can sense and migrate toward higher concentrations of SDF-1. This chemotaxis allows tumor cells to move directionally through brain tissue, often toward vascularized areas, facilitating invasion (82). Additionally, HIF-1α promotes angiogenesis via *VEGF* expression and contributes to immunosuppression by increasing nitric oxide synthases (iNOS, NOS2) activity in myeloid cells and recruiting regulatory T-cells through VEGF–neuropilin-1 signaling. All of these transcriptional effects collectively create a TME optimal for glioma expansion, not only by fueling metabolic needs but also by orchestrating the structural and immunological landscape around the tumor allowing it to thrive (**Figure 4**).

### 2.6 Mitochondrial metabolism in GBM

**Figure 5:**
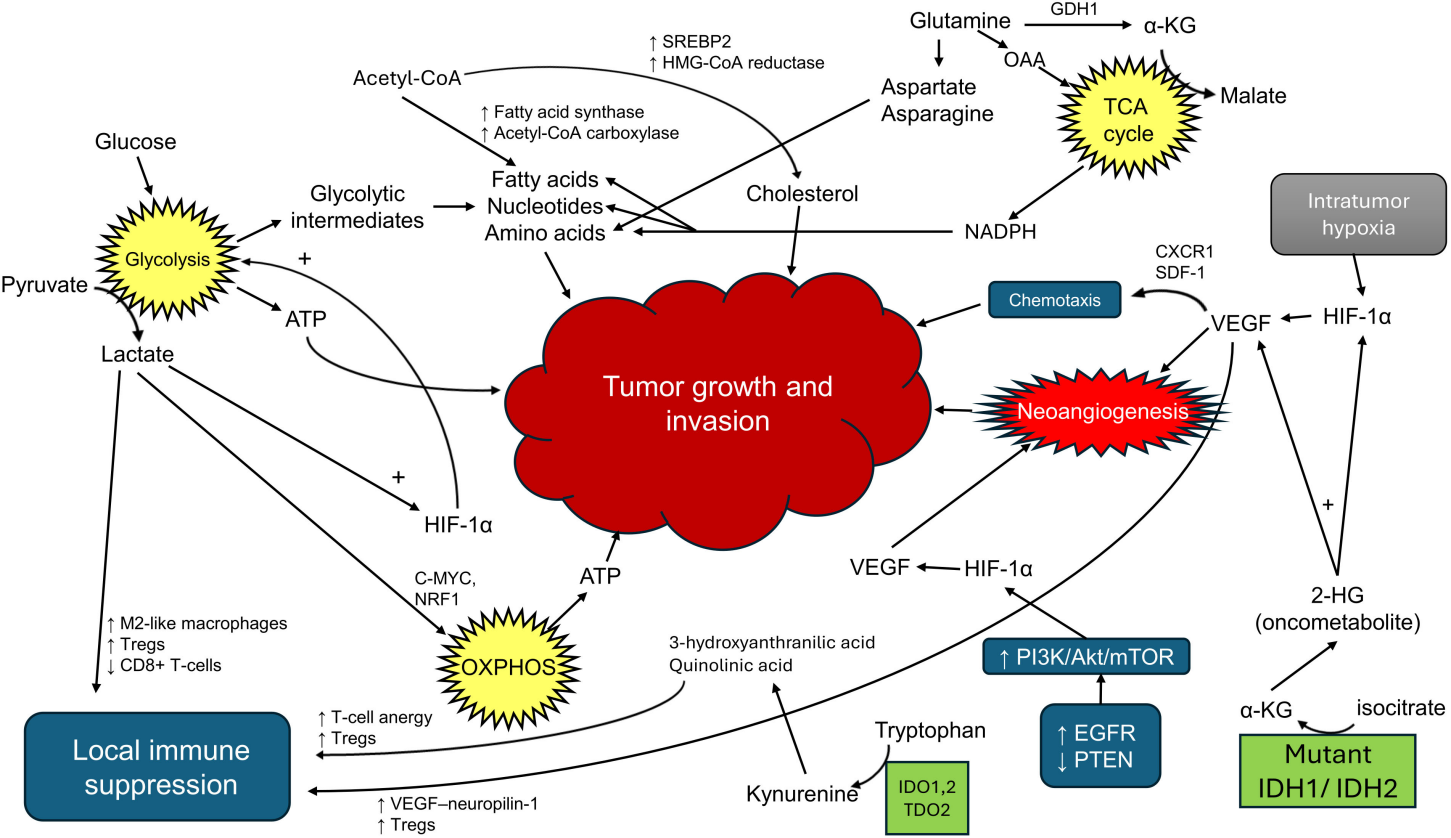
Overview of key molecular and metabolic pathways in GBM.

While many GBMs rely heavily on glycolysis, a distinct metabolic phenotype characterized by mitochondrial dominance has been increasingly recognized. This subtype demonstrates elevated OXPHOS activity and increased mitochondrial gene expression, reflecting a reliance on mitochondrial metabolism rather than glycolysis for energy production (83). Deuterium metabolic imaging has confirmed this functional distinction, showing that tumors within this subtype exhibit enhanced oxidative metabolism *in vivo* compared to their glycolytic counterparts (84). These findings underscore the therapeutic potential of targeting mitochondrial metabolism in the OXPHOS-driven mitochondrial subtype GBMs, a strategy that may differ from interventions aimed at glycolytic-dominant subtypes of GBM. This subtype of GBM exemplifies the broader metabolic plasticity of GBM, an adaptability that is responsible for its heterogeneity and therapeutic resistance. Recognizing and characterizing this metabolic flexibility opens avenues to tailor therapeutic strategies toward both glycolytic and mitochondrial subtypes (**Figure 5**).

## 3 Metabolic targeting strategies in GBM

### 3.1 Ketogenic diet as a metabolic therapy

Recently, the ketogenic diet (KD) has attracted considerable scientific attention as a potential adjuvant therapy for GBM (among other cancers) alongside standard treatment (85, 86). Although several specific variations of the KD exist (e.g. modified Atkins diet, medium-chain triglyceride diet), the primary goal of this general dietary pattern is to induce ketone body production as a primary source of energy via consumption of a higher ratio of fats to non-fats (87). Ketone bodies such as β-hydroxybutyrate (BHB) and acetoacetate are primarily produced in the liver from fatty acids, and are transported to body tissues to serve as a substrate for mitochondrial ATP production (88). This allows for efficient energy production in the absence of significant dietary carbohydrate content, while blood glucose is maintained at physiological levels via increased hepatic gluconeogenesis. Several mechanisms have been identified in the literature that provide a hypothetical basis for potential therapeutic effects of the KD in cancer, such as modulation of metabolic (89, 90), inflammatory (91, 92), and epigenetic pathways (93). As discussed further in this review, neoplastic cells often exhibit unique metabolic functioning, particularly in their preferential shift towards aerobic glycolysis for primary energy production. Mitochondrial functioning also appears to be modified in these cells, with a shift from production of ATP via aerobic metabolism to the production of ROS and precursor molecules for protein, lipid, and nucleic acid synthesis (94). Relative metabolic inflexibility can develop as a result, with increased reliance on glucose as an energy source and decreased capability of switching to alternative sources such as ketones (95, 96). This is thought to create a vulnerability in neoplastic cells to the relatively ketone-rich, glucose-deficient environment created by the KD. Mouse models have demonstrated that via increased oxidative stress and metabolic modulation, the KD can enhance sensitivity of glioma cells to radiation and chemotherapeutic agents while protecting healthy cells (97).

The most prominent ketone body produced by the body during ketosis, β-hydroxybutyrate (BHB), has been shown to modulate several inflammatory signaling pathways. Activity of the NLRP3 inflammasome has been shown to be directly inhibited by BHB via decreased K^+^ efflux and reduced ASC speck formation, leading to decreased production of IL-1β and IL-18 in human monocytes and *in vivo* models (98). It has also been demonstrated that BHB inhibits NF-κB pathways as well as histone deacetylases (99), thereby promoting hyperacetylation of histones and altering DNA transcription (100). These changes reduce production of proinflammatory cytokines such as IL-17 and promote anti-inflammatory functions in immune cells, including microglia (101). Other models in neurons have shown that BHB improves the efficiency of mitochondrial respiration by increasing the ratio of oxidized-to-reduced nicotinamide adenine dinucleotide (NAD^+^/NADH), thereby leading to a decrease in reactive oxygen species and the blunting of cell death induced by glutamate excitotoxicity (102, 103). Clinical data have demonstrated that the KD has a modest effect on markers of systemic inflammation, with an overall trend indicating significant decreases in C-reactive protein (CRP) but less meaningful impacts on IL-6 signaling (104).

Immunotherapy remains one of the most rapidly evolving and exciting areas in oncological research. However, current evidence demonstrates limited clinical utility for available immunotherapeutics in GBM, due to a highly immunosuppressive TME and metabolic characteristics that impair anti-tumor immune response. GBM cells metabolize glucose, glutamine, lipids and tryptophan to create local nutrient competition and immunosuppression (105), leading to T-cell exhaustion and expansion of Tregs and immune-suppressive M2-like macrophages (106). GBM cells increase conversion of tryptophan to kynurenine via upregulation of indoleamine 2,3-dioxygenase (IDO1/IDO2) and tryptophan 2,3-dioxygenase (TDO2), depleting local levels of tryptophan and impairing T-cell functioning (107, 108). Further downstream metabolites of the kynurenine pathway, including kynurenine itself, 3-hydroxyanthranilic acid, and quinolinic acid, have been shown to directly induce T-cell anergy, apoptosis, and regulatory T-cell differentiation (109). Quinolinic acid also modulates macrophage functioning, and drives immune tolerance through N-methyl-D-aspartate (NMDA) receptor and peroxisome proliferator-activated receptor gamma (PPARγ) signaling (110). Therapeutic targeting of the kynurenine pathway remains an active area of research.

Preclinical studies suggest that the ketogenic diet may beneficially modulate the GBM immune microenvironment by enhancing activation of CD8+ and CD4+ T-cells, as well as natural killer (NK) cells. It has also been shown to reduce expression of immune inhibitory receptors such as programmed cell death protein 1 (PD-1) and CTLA-4 on CD8+ T-cells, thereby contributing to anti-tumor immune function (111). However, in contrast to these findings, one mouse model actually found a 50% increase in M2-like macrophages with implementation of the KD, theoretically creating an immunosuppressive effect that could attenuate therapeutic benefit (112). It is important to note that there is currently much work to be done in the translation of these preclinical findings to the clinical setting. There is unfortunately no human data to date examining whether the ketogenic diet improves the efficacy, safety, or outcomes of immunotherapy in patients with GBM, either alone or in combination with standard treatments such as radiation therapy and temozolomide chemotherapy.

Despite the encouraging mechanistic findings from preclinical studies as discussed above, the existing clinical data examining the efficacy of the KD in the treatment of GBM remains limited and largely preliminary. Results from the most recent phase one trial with 17 participants demonstrated that a supervised KD was well tolerated over a 16-week period alongside standard-of-care radiation and temozolomide chemotherapy treatment, with no serious adverse events and stable or improved quality of life and cognitive function (113). The median progression-free (PF) and overall survival (OS) rates were 12.5 months and nearly 30 months respectively, but these outcomes did not reach statistical significance, and the study was not sufficiently powered for efficacy endpoints. Noteworthy adverse effects of the KD in this study included loss of appetite, flu-like symptoms, constipation, and fatigue. Other small case series and systematic reviews similarly reported safety and feasibility, with some potential evidence of improved symptom control and disease stability, but did not show conclusive survival benefit (95, 114, 115). The only randomized clinical trial to date, ERGO2, assigned 50 patients to either a calorically restricted KD with intermittent fasting or a calorically unrestricted diet while undergoing reirradiation for recurrent malignant glioma. While ketosis was reliably induced and the intervention was well tolerated by participants overall, there was no significant improvement in PF or OS compared to a standard diet (116). Explorative analysis of these data suggested that lower glucose levels (<83.5 mg/dL) at certain timepoints while receiving the KD may have been associated with better outcomes, but this has yet to be investigated further.

### 3.2 Targeting glycolysis

One of the ways by which GBM cells establish a metabolic advantage is by modulating glycolytic enzymes in favor of the Warburg effect, particularly those catalyzing the irreversible, rate-limiting steps of the process. These include hexokinase 2 (HK2), pyruvate kinase (PK), and phosphofructokinase-1 (PFK-1). Aberrant activity of these glycolytic enzymes has been observed in various cancer cell lines, notably GBM. We will first discuss how each enzyme is implicated in the Warburg effect and subsequent tumor proliferation, then cover novel inhibitors that have been developed in an effort to attenuate GBM cells’ metabolic advantage.

The first regulatory enzyme we will cover is HK2, which catalyzes the conversion of glucose to glucose-6-phosphate. By facilitating the first step in glycolysis, HK2 is responsible for promoting cell transition to the Warburg effect, which allows cancer cell lines to undergo aerobic glycolysis and utilize the metabolic advantages discussed earlier. Additionally, HK2 prevents Cytochrome C release from the mitochondria, thereby inhibiting apoptosis (76). Indeed, previous mRNA analyses demonstrated a significant link between HK2 overexpression and highly glycolytic malignant tumors. This was proven by experiments where knockout of HK2 was shown to inhibit aerobic glycolysis and induce apoptosis (76). As tumor cells grow, their core becomes hypoxic and under normal circumstances, should become necrotic. Therefore, increased cell death would be predicted in the necrotic core of tumors. This was not the case, however, in GBM tumors. Interestingly, PCR analyses of GBM core cells showed high expression of HK2. While HK2 is expressed at varying levels in skeletal and adipose tissue, its levels are negligible in a healthy brain, where HK1 is the predominant isoform. This indicated that GBM cells could be overexpressing HK2 in order to confer a metabolic advantage. This adaptation was confirmed in other studies, where HK2 levels were measured to be about one-hundred times greater in GBM cells relative to normal cells (117). Furthermore, experimental knockout of HK2 *in vivo* resulted in significant decrease in tumor size, vasculature, and lactic acid. Combined, this shows that HK2 may be a potent activator of glycolysis and tumor proliferation in GBM, making the enzyme a potential target in chemotherapies.

Another important driver of tumor metabolism is PFK-1. PFK-1 catalyzes the irreversible phosphorylation of fructose-6-phosphate to fructose-1,6-bisophosphate, governing the glycolytic flux. PFK-1 exists as three isoforms depending on tissue location, platelet-type (PFKP), liver-type (PFKL), and muscle-type (PFKM) (118). Studies have shown substantial changes in the expression of these isoforms in malignant tumors. Similar to the other rate-limiting glycolytic enzymes, PFK-1 overexpression causes increased activity of glycolysis regardless of oxygen level (Warburg effect), which is the first way in which tumor cells have utilized the enzyme for continuous growth. In the case of GBM cells, its overexpression is achieved via AKT-mediated phosphorylation. Phosphorylation of PFKP inhibits TRIM21 E3 ubiquitin ligase-dependent activation, thereby increasing PFKP stability by preventing its ubiquitylation and degradation. This results in increased PFK expression and promotion of aerobic glycolysis. Further studies are needed to elucidate non-canonical function of PFK-1 in tumor development (119).

The final and perhaps best studied enzyme implicated in glycolytic modulation of GBM cells is pyruvate kinase (PK). PK is the final rate-limiting enzyme of glycolysis, catalyzing the conversion of phosphoenolpyruvate (PEP) to pyruvate with the concomitant generation of ATP. PK is the key regulatory enzyme in glycolysis, whereby, depending on cellular energy demands and metabolic signals, it either increases or decreases the rate at which cells perform glycolysis. Different PK isoforms are expressed in various tissues, each with unique regulatory properties. For example, the PKM1 isoform is exclusively found in tissues with high catabolic activity, like the heart, brain, and muscles, while PKM2 is generally present in all proliferative and cancer cells. Importantly, PKM2’s prevalence in rapidly dividing cells makes it a critical point of regulation as it is implicated in manipulating the properties of aberrant glucose metabolism in cancer cells (120). PKM2 overexpression has been consistently observed in gliomas and GBM, with higher levels correlating with tumor grade and poorer prognosis (121–124).

**Figure 6:**
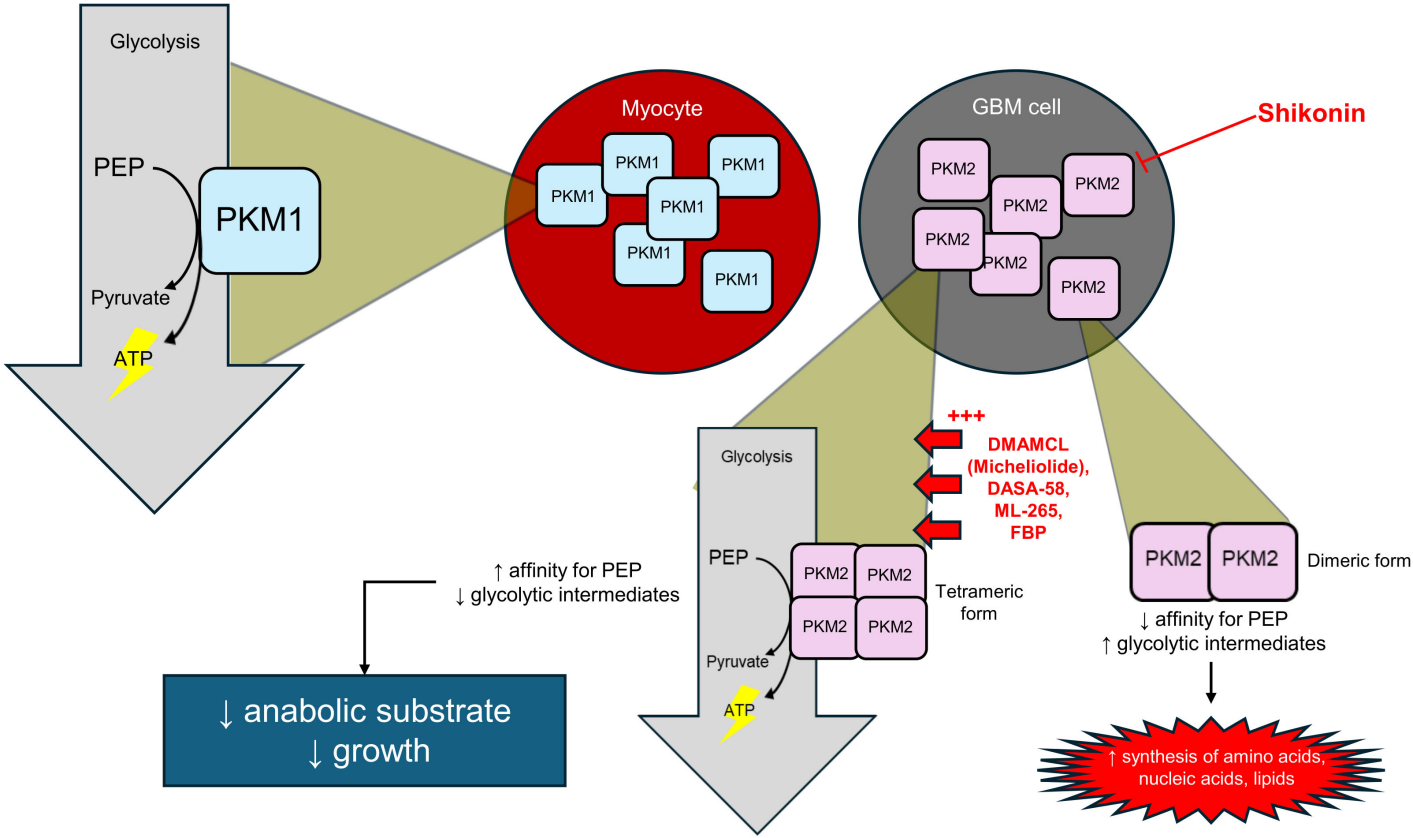
PKM2-targeted strategies in GBM: inhibition or tetramer activation to disrupt tumor metabolism.

PKM2 is unique in that it assumes a dual role for cancer proliferation: in its tetrameric form, it drives ATP production, providing energy for growth; in dimeric form, it redirects glycolytic intermediates towards anabolic pathways that support rapid proliferation. This flexibility enables cancer cells to balance energy production and proliferative phases for the most efficient growth. Because PKM2 activation enhances glucose uptake, increases lactate production, and inhibits autophagy, its overexpression plays a crucial role in modulating the TME and driving tumor progression (125). Regulation of PKM2, and PKs in general, is achieved at the level of its quaternary structure by covalent modification—including phosphorylation, acetylation, and oxidation—which influence its oligomeric state and metabolic output. For example, phosphorylation of Tyr105 disrupts tetramer formation and reduces catalytic activity, while oxidation of Cys358 diverts glucose flux into the pentose phosphate pathway. Therapeutic strategies targeting PKM2 aim to manipulate these regulatory mechanisms, either by stabilizing the active tetramer to force maximal glycolytic flux (PK activators), thereby starving the tumor of anabolic building blocks, or by inhibiting PKM2 activity to starve the tumor of energy (PK inhibitors) (**Figure 6**) (126).

Given GBM’s reliance on reprogrammed metabolic pathways which confer sustained growth and survival, targeting these aberrant processes has emerged as a potential therapeutic approach. The first of these approaches targets the Warburg effect. This metabolic shift from OXPHOS to glycolysis is characteristic of many cancers, including GBM, and provides a substantial survival advantage as it protects cancer cells from the hypoxic TME and the cytotoxic effect of oxidative damage and mitochondrial-mediated apoptosis. To disrupt the aerobic glycolysis characteristic of GBM cells, several inhibitors have been developed against key enzymes responsible for this metabolic shift, namely, pyruvate kinase M2 (PKM2) and pyruvate dehydrogenase kinase (PDK).

The first of these strategies we will discuss here is shikonin, an inhibitor of PK. In preclinical GBM models, shikonin, a naphthoquinone derivative, reduced PKM2 phosphorylation at Tyr105, suppressing aerobic glycolysis and impairing tumor growth (127). Though still in its preclinical stage, shikonin has been shown to inhibit PKM2 at concentrations that resulted in over 50% inhibition without affecting PKM1. In addition, it was found to inhibit glucose consumption and lactate release in MCF7 and A549 tumor cells (breast and lung cancer cell lines, respectively). Moreover, a study by Huang et al. (128) found that shikonin not only inhibits PKM2 phosphorylation but also blocks translocation of the enzyme to the nucleus (128). In effect, this prevents tumorigenesis as traditionally, PKM2 can interact with nuclear proteins such as β-catenin to activate genes involved in cell proliferation and Warburg effect activation (129). This effect was recognized across multiple studies and cell lines, and no indication as of yet precludes this mechanism of action from working on GBM cells. These combined effects of shikonin give it promise to mature into a potential anticancer drug used for GBM (130).

While inhibitors of PKM2 reduce glycolysis and the Warburg effect, activators do the opposite, constitutively forcing PKM2 into its tetrameric form and starving cancer cells of the anabolic intermediates they were otherwise building for tumor proliferation. Both PKM2’s highly active tetrameric form and its less active dimeric form are implicated in tumor growth, but the dimeric form was found to predominate in GBM cells (131). Therefore, activators capable of promoting the tetrameric form of PKM2 and increasing PK activity could be a potential therapy for GBM. Various PKM2 activators were developed with this goal, including dimethylaminomicheliolide (DMAMCL), DASA-58, ML-265, and 1,5-2H-pyrrole-dione derivatives (132). Guo et al. (131) developed the small molecular compound DMAMCL as a potent PKM2 activator, which has been used in clinical trials for recurrent GBM in Australia. Micheliolide (MCL), which is the active component of the compound, binds to monomeric PKM2 and promotes its tetramerization, increasing the activity of the PK in GBM cells. Moreover, GBM cells treated with DMAMCL were found to have decreased glycolytic intermediates of lactate and glucose-6-phosphate, further demonstrating an inhibitory effect on glycolysis. In essence, treatment with DMAMCL suppresses the proliferation of GBM cells and inhibits tumor growth (131).

Anastasiou et al. (133) demonstrated that both DASA-58 and ML-265 modulate the glycolytic cascade, leading to inhibited tumorigenesis in a xenograft model. These molecules bind to the dimer-dimer interface of PKM2, promoting its tetramerization and thereby attenuating tumor growth. The above activators are appropriately suited as potential anti-tumorigenic compounds not only because they activate PKM2 and attenuate the Warburg effect, but also because they are selective in their binding to PKM2, sparing PKM1 from constitutive activation. This selectivity is due to the activators’ heterocyclic core, which results in high affinity to the enzyme. However, this offers a disadvantage because the presence of heterocyclic cores makes the compound insoluble in aqueous environments, posing a significant limitation in its efficacy. Efforts need to be made towards making water-soluble analogs of PKM2 activators (120). Naturally occurring ligands have been found to exhibit similar effects on PKM2, specifically, fructose 1,6-bisphosphate (FBP). Rathod et al. (120) explain how FBP binding to PKM2 forces the PK into its active, tetrameric state, which favors PEP recognition at the active site, enhancing enzymatic activity. In the same way as the artificial molecules, FBP can forcefully induce glycolysis via PKM2 activation, preventing cancer cells from redirecting glycolytic intermediates towards making the building blocks necessary for tumor proliferation (120). Indeed, this will inhibit tumor growth and cause a decrease in size, as discovered in mouse models.

Another potent modulator of the Warburg effect is pyruvate dehydrogenase kinase (PDK). Because cancer cells like those of GBM utilize aerobic glycolysis, targeting the intersection of mitochondrial metabolism and cell surface mechanisms may serve as a possibility for reversing the Warburg effect. In normal cells, pyruvate dehydrogenase (PD) is responsible for converting pyruvate into acetyl-CoA, feeding the TCA and OXPHOS in mitochondria. As discussed above, it is in cancer cells’ best interest to forego this process, preventing OXPHOS from taking place and instead using aerobic glycolysis and lactate production as energy sources. This is achieved by PDK utilization. PDK phosphorylates PD, which inactivates the enzyme. This prevents the conversion of pyruvate to acetyl-CoA, inhibiting OXPHOS from progressing. The metabolic shift from the TCA cycle/OXPHOS to aerobic glycolysis is tightly controlled. Indeed, PDK activity is upregulated in multiple cancer types and has been associated with tumor aggressiveness, proliferation, anti-apoptotic effect, and therapy resistance (134). The mechanism by which PDK activity is upregulated in cancer cells comes as a result of downstream activation by HIF1a, which is induced by the hypoxic TME (134). Therefore, efforts have been made to develop PDK inhibitors, which will cease phosphorylation of PD and force OXPHOS to occur, denying cancer cells their ability to enjoy the benefits conferred to them by aerobic glycolysis.

One of the most well-characterized inhibitors of PDK is dichloroacetate (DCA), a small (150 Da), water-soluble molecule long used in the treatment of metabolic disorders such as lactic acidosis, inborn errors of mitochondrial metabolism, and diabetes (135). More recently, it has been repurposed as an anticancer drug, which is now in clinical trials. DCA acts as a small molecule inhibitor of PDK, maintaining PD in its unphosphorylated, active form and facilitating TCA/OXPHOS. Additionally, DCA readily crosses the blood-brain barrier and has been shown to activate PD and reverse the Warburg effect in glioma cells (136). Michelakis et al. (137) experimented with DCA in GBM cells using tumor tissue samples from 49 patients and measured mitochondrial membrane potential (index of mitochondrial function). They found that potential increased, indicating increased mitochondrial function (OXPHOS), while not affecting normal brain tissue. They then treated five recurrent GBM patients with DCA and found that three of the five experienced tumor regression on imaging. Efficacy of the drug was also proven by Jiang et al. who demonstrated an increase in cell death percentage from a sample of GBM stem cells treated with DCA. Finally, Morfouace et al. (138) confirmed that DCA decreases glycolytic metabolism through PDK inhibition in rat glioma cancer stem cells but not in rat neural stem cells. These are a collection of multiple studies that have demonstrated DCA to be a selective inhibitor of PDK and a truly promising therapeutic application for GBM. Moreover, Tataranni et al. (135) have shown that DCA can be used synergistically with other chemotherapeutic approaches to maximize the targeting of GBM cell proliferation. Firstly, DCA administration has been described to predispose tumor cells to radiotreatment, as increasing OXPHOS creates reactive oxygen species. In addition, an effective combination of DCA with paclitaxel and doxorubicin was observed via increased cell death and autophagy inhibition—effects greater than either drug alone. Interestingly, PDK overexpression is associated with chemoresistance, so it is appropriate that DCA inhibition of PDK will resensitize cancer cells to drugs (136). Overall, findings from these studies provide a strong rationale for moving DCA into clinical translational studies for cancer therapy, especially in GBM patients.

### 3.3 Targeting glutaminolysis

GBM cells utilize various metabolism reprogramming techniques to maximize energy production. While the Warburg effect is the cornerstone of malignant cells’ energy dysregulation, additional reprogramming techniques are frequently employed as well. Glucose is not the sole source of energy for cancer cells—they also utilize glutamine as an indispensable substrate in tumor cell metabolism, supporting bioenergetics and biosynthesis (139). This phenomenon, known as “glutamine addiction,” refers to cancer cells’ ability to favor sustained glutamine metabolism, not only providing energy, but also supporting the biosynthesis of the nucleotides, proteins, and lipids necessary for aberrant tumor proliferation. To provide these effects, glutamine is metabolized through glutaminolysis within the mitochondria, converting it to glutamate and TCA intermediary α-KG. Being the most abundant amino acid in plasma, glutamine serves as a crucial substrate in tumors due to its role as a carbon and nitrogen donor for fueling growth-promoting pathways. Similarly to how HIF-1α upregulates glycolytic enzymes, its increased expression in tumor cells also induces glutaminolysis by directly or indirectly activating glutamine transporters (139). Specifically, an upregulation of glutaminolysis was observed in gliomas, noting increased levels of extracellular glutamate in affected brains compared to normal (54). Buckingham et al. (140) confirmed glutamate release from glioma cells *in vivo* by measuring levels after glioma implantation into rat brains. Trejo-Solis et al. (54) also discuss how malignant gliomas exhibit increased glutamine uptake and consumption, noting increased levels of intratumoral glutamine relative to normal brain tissue (54). This effect was observed in GBM xenografts in mice as well as by magnetic resonance spectroscopy in GBM patients. As a response to HIF-1α signaling, cancer cells upregulate glutamine intake and subsequent metabolism by increasing transporters on the plasma membrane, allowing more glutamine into the tumor. Glutamine is transported into the cell by multiple solute carrier (SLC) type transporters, including SLC1A5, SLC7A5, and SLC7A11 (139). Once inside the cell, it is catabolized by GLS, forming glutamate and ammonia in the process. Next, glutamate is metabolized by transaminases or GDH1, forming α-KG. α-KG is then carboxylated to produce isocitrate, which is converted to citrate. Finally, ATP-citrate lyase (ACLY) uses the citrate carbon to produce acetyl-CoA, allowing TCA cycle progression and ATP production (139). Understanding this pathway, in addition to the fact that glutamine metabolism is upregulated in GBM cells, has prompted discoveries of various metabolic modulators, including GLS and GDH inhibitors, targeting the energetic advantage this pathway provides.

In the study of glioma cell GLS inhibitors, three pharmacotherapies have been developed: compound 968, bis-2-(5-phenylacetamido-1,2,4-thiadiazol-2-yl) ethyl sulfide (BPTES), and CB-839. It is important to mention that glutaminase C (GAC), a splice variant of GLS1, is more catalytically active and is the isoform upregulated in a variety of cancers (141), making it the principal point of therapeutic targeting (142). Originally identified by Stalnecker et al. (143), compound 968 (C968), also known as bromo-benzophenanthridinone, is a lead compound that preferentially inhibits GAC and prevents oncogenic transformation. Various studies have tested compound 968’s effect on multiple cancer cell lines, including ovarian and non-small cell lung cancer, and found significant reduction in tumor cell proliferation (144). In 2015, Tanaka et al (145). showed that C968 significantly suppressed GBM proliferation through inhibition of GAC, as measured by decreased glutamine uptake and decreased production of glutaminolysis byproducts. The drug was also found to potentiate the effects of mTOR-targeted treatment (a different treatment approach to be used as a combination therapy) (144). Interestingly, a more recent study by Koch et al. (146) in 2020 found that compound 968, even at high concentrations, did not affect GAC enzymatic activity. Consensus surrounding C968 is that while it has not been proven to directly inhibit GAC in GBM cells, it does enhance the anti-GBM effects of mTOR inhibition, working as a potent therapeutic in that regard. There is a point of contention amongst the literature, and more studies need to be carried out to find the exact effect C968 has on GAC and subsequent GBM cells proliferation (146).

Another therapy targeted at inhibiting glutaminase in GBM is the uncompetitive allosteric inhibitor BPTES, which stabilizes GAC in an inactive tetrameric state. Indeed, BPTES was found to inhibit GLS and slow GBM growth as measured by decreased α-KG levels, acting as an effective pharmacological suppressor of tumor cell proliferation (147). Additionally, BPTES has been tested in human GBM cell lines with the IDH1 mutation and was found to exhibit profoundly decreased proliferation of the tumor. Unlike C968, BPTES study results indicate that it has the potential to be effective in the treatment of tumors with elevated glutaminolysis, including GBM (148). However, while BPTES is a potent inhibitor of GAC, its low solubility makes it difficult to deliver *in vivo*. For this reason, a clinically tested derivative, CB-839, was developed (149). CB-839, also known as Telaglenastat, is the next-generation glutaminase inhibitor optimized from the older BPTES. Like BPTES, CB-839 binds to the allosteric pocket of tetrameric GAC, stabilizing the inactive conformation. Eventually, this starves cancer cells of α-KG, NADPH, and nucleotide synthesis. Additionally, CB-839 has better oral bioavailability than BPTES, making it a stronger option moving forward (150). Currently, CB-839 is being tested in multiple phase I and II clinical trials for cancers with high glutamine dependence, including triple negative breast cancer, renal cell carcinoma, and leukemias and lymphomas. In animal models, it has been found to be an excellent suppressor of GBM cell proliferation, an effect that can be reversed by supplementation with α-KG. Jiminez et al. (151) found that CB-839 inhibited GLS in three different GBM cell lines, which was reflected in strong, dose-dependent antiproliferative effect on the cancer cells (151). This indicates that CB-839 is indeed a potent disruptor of glutaminase in cancer cells (151). While CB-839 shows promising anti-GBM effects, there are no current clinical trials in GBM patients. Further studies need to be completed in order to draw more accurate conclusions about the drug’s efficacy in GBM.

Currently, most research is targeted at developing GLS inhibitors as a potential therapeutic approach to GBM. However, new studies have found that inhibiting GDH is another possible point of regulation. As we discussed earlier in this section, α-KG is formed from glutamate by GDH. It is then used in the TCA to generate NADH for ATP production as well as to serve as a precursor in protein synthesis. Excess α-KG formation in cancer cells causes a higher influx of the intermediate into the TCA cycle, further activating it (152). Therefore, inhibitors effective at targeting GDH and subsequent α-KG generation can potentially attenuate tumor proliferation. Previous studies have shown that targeting mitochondrial GDH, which catalyzes the conversion of glutamate to α-KG, has inhibited the proliferation and migration of cancer cells. Specifically, a compound called epigallocatechin gallate (EGCG) serves as a strong inhibitor of GDH1 and has proven its efficacy by suppressing the proliferation of glioma cells. Another compound, R162, a purpurin analog and inhibitor of GDH1, also demonstrated this effect *in vitro* and in patient-derived xenograft mouse models (68). This is still a new area of research with regards to glutaminolysis modulation, and more studies are necessary for refinement of GDH inhibitors.

### 3.4 Targeting mitochondrial dysfunction

As we have discussed so far, GBM cells display remarkable metabolic flexibility, utilizing glycolysis and glutamine-driven oxidative metabolism to fuel uncontrolled proliferation. Each of the various metabolic targets discussed so far aim to inhibit a single enzyme in a metabolic pathway, preventing tumor cells from accessing the energetic and anabolic demands required for growth. A different approach to this inhibition is to target the mitochondria itself, which is where such pathways take place. Being essential for ATP production, biosynthetic precursor creation, redox balance, and apoptosis regulation, disrupting mitochondrial function can both starve GBM cells metabolically and force them into apoptosis.

Recall that mitochondria are made of two membranes, inner and outer. The inner membrane is the site of OXPHOS, while the outer membrane controls energy flux and exchange of metabolites through one of multiple isoforms of voltage-dependent anion channel 1 (VDAC1). VDAC1 serves as the metabolic connection between the inner mitochondria and the cytosol. It allows for entry of metabolites, ions, nucleotides, and calcium, among other cellular components. Additionally, it regulates the release of pro-apoptotic proteins from the mitochondria and interacts with anti-apoptotic proteins to prevent its oligomerization and channel formation, thus blocking apoptosis. Therefore, manipulating VDAC1 gives us the ability to not only regulate the flux of metabolites into the mitochondria, but also predispose it to activating apoptosis in cells. It is well documented in the literature that VDAC1 is overexpressed in many cancer types, including GBM. Shteinfer-Kuzmine et al. (68) led the way in studying VDAC1 inhibitors in GBM cell lines and found that competitive peptide analogs successfully altered VDAC1 activity and caused remarkable tumor growth inhibition. The two peptide analogs used in the study include Tf-D-LP4 and D-ΔN-Ter-Antp. D-ΔN-Ter-Antp is a 16 residue-long sequence fused to a VDAC1-N-terminal sequence. Tf-D-LP4 is a penetrating peptide comprised of a VDAC1-derived sequence fused to human transferrin receptor (hTfR)-recognition sequence, which is highly expressed in many cancers. In this way, the peptide analog displays selectivity to cancer cells lines and is taken directly to GBM cells *in vitro*. Once inside GBM cells, Tf-D-LP4 exerts various effects on enzymes involved in ATP generation as well as apoptosis initiation, depleting the former and promoting the latter. Firstly, D-ΔN-Ter-Antp and TF-D-LP4 were found to significantly decrease membrane permeability of cancer cell mitochondria, resulting in an 80% decrease in cellular ATP (153).

These results show that peptide treatment dramatically decreased cell energy production. Additionally, D-ΔN-Ter-Antp and TF-D-LP4 were found to induce apoptosis in U-87MG (GBM cell lines) by inducing cytochrome c release from the mitochondria. Shteinfer-Kuzmine et al. (68) recognized apoptosis in 63% to 74% of GBM cells treated with the peptide analogs. The results of the combined effects of D-ΔN-Ter-Antp and TF-D-LP4 were reflected via *in vivo* studies showing a 45% decrease in tumor size in mice with GBM—marking a significant decrease in tumor proliferation (154). Another approach, taken by Arif et al. (155), found that silencing GBM cell VDAC1 with interfering RNA can also stunt tumor growth through a multifaceted mechanism of action like the peptide analogs described above. His team also showed that treating U-87 cell lines with itraconazole, an antifungal, reduced channel conductance across lipid bilayers and decreased membrane potential. Similar to the peptide analogs, the decrease in membrane potential inhibited ATP production, as reflected in a ~60% decrease in U-87 tumor volume in xenografts treated with itraconazole (155). Through direct manipulation of ATP generation and promotion of apoptosis, VDAC1 inhibitors show promising results in their ability to inhibit tumor cell proliferation, proving potential for replacing several anticancer drugs that separately target angiogenesis, proliferation, or metabolism (154).

## 4 Therapeutic approaches to reverse hypoxia in GBM

### 4.1 Hypoxia-activated prodrugs: the case of evofosfamide

While inactive in oxygen-rich normal tissues, hypoxia-activated prodrugs (HAPs) leverage the low-oxygen environment of a tumor (156–158). Intracellular reductase will reduce the prodrug under hypoxic conditions, activating it and releasing a potent cytotoxic agent that crosslinks and damages DNA (157–159). Furthermore, the active metabolite can exhibit a “bystander effect” that allows for cytotoxic spread beyond the hypoxic region and onto adjacent normoxic cells (157, 159). This mechanism is evident with evofosfamide (TH302), a second-generation hypoxia-activated nitroimidazole prodrug (157, 160). Evofosfamide’s nitroimidazole component is reduced in hypoxic tumor regions by intracellular reductase, releasing cytotoxic alkylating agent bromo-isophosphoramide mustard (Br-IPM) (157, 159, 160). The selectivity for hypoxic zones makes HAPs like evofosfamide a viable option for treating GBM (156, 157).

Evofosfamide has also been studied in clinical trials focusing on recurrent GBM cases refractory to bevacizumab (Bev) (159). Bev is an anti-angiogenic agent that induces tumor hypoxia, which as a result would synergistically provide the ideal conditions for activating HAPs (159, 161). Phase I of this trial (NCT02342379) found that with up to a maximum dose of 670 mg/m² of Bev and evofosfamide combined therapy, patients with recurrent GBM had tolerated it well, with safety and preliminary efficacy data showing a 17.4% overall response rate and 60.9% of patients with disease control (159). Using Dynamic Susceptibility Contrast (DSC)-magnetic resonance imaging (MRI) and fluoromisonidazole (18F-FMISO) positron emission tomography (PET) imaging, phase ll of the study additionally examined the role of hypoxia as a biomarker for therapeutic efficacy in patients with Bev-refractory GBM being treated with the combined Bev and evofosfamide therapy (161). 18F-FMISO is retained in hypoxic cells, making it a non-invasive method to monitor tumor hypoxia (161). Furthermore, DSC-MRI is used to attain perfusion parameters like standardized relative cerebral blood volume (SrCBV) and time to maximum value of residue function (Tmax) (161). A significant inverse correlation was found in these treated patients, where decreased hypoxic volumes were related to longer OS and PF survival (161). Higher srCBV and lower Tmax were associated with lower OS, indicating that these features could be useful in evaluating treatment and guiding clinical considerations (161). The study suggests potentially improved outcomes for patients with Bev-refractory GBM that are treated with evofosfamide to reduce hypoxic volume in combination with Bev. Since this combined therapy can be administered safely at full recommended doses, it warrants further investigation with a larger population to understand its clinical use (161).

### 4.2 Hyperbaric oxygen therapy

Hyperbaric oxygen therapy (HBOT) has been another approach that works to increase oxygen supply while reducing hypoxia, inflammation, and edema within the TME (156, 160, 162, 163). By being in a hyperbaric chamber and breathing in 100% oxygen at high atmospheric pressures (>1 ATA), HBOT sensitizes GBM cells to therapies like radiotherapy and chemotherapy (160, 162, 163). During radiotherapy, DNA damage and cell death occur as a result of Reactive Oxygen Species (ROS) formation caused by the effects of radiation on oxygen (163). The hypoxic regions of GBMs can cause resistance to such antineoplastic treatments like radiation due to impaired ROS formation (163). HBOT has been found in both *in vitro* and *in vivo* preclinical studies to reduce the effects of hypoxia by significantly decreasing HIF-1α/HIF-2α expression at the transcriptional and translational levels (162–164).

HIF-1α and HIF-2α are transcription factors that have a role in the hypoxia-signaling pathway and have been linked to increased proliferation, invasion, and therapy resistance in GBM (162–164). HIF-1α and HIF-2α become stabilized under hypoxic conditions, where they initiate a coordinated transcriptional program that enables tumor cells to survive in low-oxygen environments. Rather than resolving hypoxia, these factors activate the expression of genes involved in anaerobic glycolysis (e.g., GLUT1, HK2, LDHA), angiogenesis (e.g., VEGF), invasion (e.g., MMP2, MMP9), and stem cell maintenance. This metabolic and phenotypic reprogramming enhances proliferative and invasive capacity while contributing to therapeutic resistance in GBM. Thus, HIF-1α and HIF-2α act as adaptive mediators of hypoxia rather than resolving it, making them central to the malignant progression of glioblastoma. By evaluating the roles of HIF-1α and HIF-2α on GBM, we can understand the mechanisms driving outcomes in HBO therapy use for GBM. Wang et al. (2025) helped foster this connection, where they reported downregulated HIF-1 signaling pathways, cell metabolism, cell cycle activity, and apoptosis in HIF-1α knockout cells compared to downregulation of stemness pathways and cell cycle activity in HIF-2α knockout cells. Single HIF-1α or HIF-2α knockout cells were also noted to have an increased apoptosis rate that was even more significant in the simultaneous HIF-1α and HIF-2α knockout group when compared to the control group (164). These findings suggest HIF-1α and HIF-2α synergistically regulate GBM malignancy and can act as a target to reduce hypoxia through therapies like HBO therapy.

Wang et al. (2025) demonstrated HBO as a potential therapy when reporting that HBOT-treated GBM cells had significantly decreased expression of HIF-1α and HIF-2α when compared to the control group, allowing for chemosensitization (164). Compared to the control group under hypoxic culture, GBM cell growth rate was found to be increased with inhibited cell invasion (164). When treated with an equal dose of temozolomide (TMZ), the HBOT group has a significantly higher apoptosis rate, significantly reduced growth rate, and more cells in G2/M + S than in G1 when compared to the control group (164). This was also reflective in their *in vivo* study, where compared to the control under normoxic conditions, the HBO group not treated with TMZ were noted to have a shorter survival time and larger tumor size and weight (164). The HBO group treated with TMZ had not only longer survival times compared to the control but also lower tumor size and weight (164). HBOT additionally reduces the expression of ATP-binding cassette subfamily G member 2 (ABCG2) through inhibiting HIF-1α-mediated pathways (162). ABCG2 is highly expressed in the hypoxic microenvironment of glioma cells, acting as a drug efflux transporter and tumor stem cell marker (162). Through HBO therapy, one can reduce HIF-1α, a transcription factor of ABCG2, to reduce ABCG2 expression (162). This makes it a therapeutic target for intervention to reduce tumor multidrug resistance and increase chemosensitivity in GBM.

HBOT has been clinically explored with conjunctive multiagent chemotherapy and radiotherapy for patients with high-grade gliomas (156, 163, 164). A Phase II trial consisting of 39 high-grade glioma patients attained a median OS of 17.2 months after receiving daily radiotherapy 15 minutes post-HBOT and multi-agent chemotherapy (156, 165). Another trial where patients were subject to an Intensity Modulated Radiation Therapy (IMRT) and TMZ-based chemotherapy with HBOT was found to have a median OS of 22.1 months (156, 163, 166). Another study subjected patients with recurrent high-grade glioma to hypofractionated stereotactic RT (FSRT) received 1 hour following HBOT (163). The pilot study reported a median OS of 10.7 months, a median PFS of 5.2 months, and a 55.5% disease control rate after HBOT-RT (163, 167). Despite some cases of acute toxicities or symptomatic radionecrosis, combined HBOT with radiochemotherapy is noted to be safe and tolerable for patients (156, 163, 164). Additionally, performing radiation within 15 minutes post-HBOT has been reported to demonstrate peak radiosensitivity of GBM cells, shining light on the vital role in the timing of administration of treatment (160). While promising, studies with multiple therapies combined with HBOT make it difficult to isolate and understand the exact contributions of HBOT alone. Therefore, further randomized studies are needed to understand HBOT in GBM before integrating it into standard clinical practice.

### 4.3 Oxygen transport agents

Agents like trans-sodium crocetinate (TSC) (C_20_H_22_Na_2_O_4_), derived from crocetin (C_20_H_24_O_4_), interact with water molecules to form a densely packed matrix that enhances oxygen diffusion into hypoxic tissue sites (156). These agents achieve this by reducing the flow resistance and density of the plasma fluid (156). Preclinical studies noted increased median survival and significantly reduced tumor growth rate and size in C6 glioma rat models treated with combined TSC and radiation therapy (RT) (156). TSC is currently in a phase III clinical trial (NCT03393000). In prior GBM clinical trial, TSC was given with concomitant radiotherapy and TMZ (156). Long-term results reported that 36% of patients who received the full dose of TSC were alive at 2 years, surpassing the 27% seen with the RT and TMZ group (156). To address TSC pharmacokinetic challenges regarding rapid peak concentration post-injection and its short half-life, a liposomal encapsulation (LEAF-4L6715) has been developed, showing promising tolerability among patients (156).

Myo-inositol trispyrophosphate (ITPP) hexasodium salt is an allosteric effector that enhances oxygen delivery to hypoxic regions with the ability to cross the BBB, making it of interest when treating GBM (156). ITPP works by reducing hemoglobin’s oxygen-binding affinity, increasing the oxygen-release capacity of red blood cells (156). Preclinical results in the literature have not been uniform; notably, a 9L-glioma rat model study reported complete cures within the combined ITPP and RT group while also observing similar results to that of the RT-only group (156). Another preclinical rat GBM model study also reported no additional effect when treated with ITPP (156). In addition to its ability to enhance oxygen delivery, ITPP acts as a tumor vascular stabilizer by activating endothelial PTEN (156).

### 4.4 HIF-1/2α inhibitors as a therapeutic target

Despite standard protocol consisting of surgical resection, radiotherapy, and chemotherapy that have remained largely unaltered since 2005, GBM’s aggressive nature to recur continues to challenge the medical community for more novel therapeutic approaches (156). GBM’s resistance to therapy and malignant abilities arises from its limited capacity for diffusion and chronic hypoxia (156, 157, 163). Such hypoxic conditions stabilize and activate HIF-1α protein through inactivation of Prolyl-4-hydroxylases (PHD) and factor inhibiting HIF-1 (FIH-1) enzymes. Once stabilized, HIF-α translocates to the nucleus, dimerizes with HIF-1β/Aryl Hydrocarbon Receptor Nuclear Translocator (ARNT), and forms a HIF transcription factor to promote cellular pathways influencing proliferation and malignant progression (39, 156–158, 164). Under normoxic conditions, HIF-α protein is destabilized by hydroxylation mediated by PHDs (158). Additionally, inhibition of transcription by blocking CBP/p300 interaction is mediated by FIH-1 hydroxylation under normoxic conditions (158). HIF-1α is further upregulated by GBM’s poor perfusion, as its atypical neovascularization contributes to a cycle that exacerbates its hypoxic environment and shields it from current therapies (39, 156, 157, 163).

Previously overlooked, direct interference with HIF-1α pathways can lead to a change in the management and outcomes of GBM treatment. OKN-007 is an agent that acts as an inhibitor of HIF-1α transcription and expression (156). EZN-2208 is another agent that targets and inhibits translation of HIF-1α mRNA (156). Likewise FDA-approved agents like Topotecan, a topoisomerase I inhibitor, were found to carry inhibitory effects on HIF-1α translation (156, 158, 160). Cardiac glucoside Digoxin has also been found to effectively inhibit translation of both HIF1α and HIF2α (160). Digitoxin was found to suppress HIF-1α in GBM stem cells with high specificity (160). Other agents like Melatonin, Curcumin, and EF-24 promote of HIF-1α degradation while agents like Acriflavine, Echinomycin, and KCN1 work to inhibit HIF’s ability to bind with their hypoxia-responsive element (HRE) domain (156). HIF-1α may also be indirectly modulated through various agents to treat GBM tumors. Traditionally used as an anti-diabetic agent, Metformin was not only found to decrease HIF-1α expression of TMZ-resistant GBM cells in combination with TMZ but also, when used alone, was able to reverse hypoxia-induced genes by reducing the oxygen consumption rate (156). HIF-1α levels can be reduced under both normoxic and hypoxic conditions through inhibiting the PI3K/AKT/mTOR pathway that modulates HIF-α mRNA translation (158). Under normoxic and hypoxic conditions, Geldanamycin acts as an inhibitor of heat shock proteins to drive proteasomal degradation of HIF-1α in a VHL-independent manner (158). By inhibiting HIF-1α’s transcriptional activity, FDA-approved proteasome inhibitors like Bortezomib can block the accumulation of proteins like CAIX, EPO, and VEGF (158).

Despite recognizing HIF-1α inhibition as a potential target to treat GBM, concerns still lie regarding ubiquitous expression in non-tumor tissues that can lead to potential systemic side effects (156). Conversely, studies suggest HIF-2α expression to be more specific for tumor tissue and to correlate with higher glioma grades when present in higher levels (156). PT2385 is one of the very few HIF-2α inhibitors investigated in preclinical models of GBM and works by preventing allosteric heterodimerization with HIF-1β when it binds to HIF-2α PAS-B domain (156). When used alone, PT2385 was found to increase the median OS of mice in comparison to the control group (156). This added benefit is not noted when combined with RT and TMZ (156).

### 4.5 Noscapine and other small-molecule inhibitors

Noscapine is a phthalide isoquinoline alkaloid that has historically been used as an antitussive agent due to its non-addictive nature (168). Its potential role for use in GBM relates to its ability to cross the blood-brain barrier (168, 169).It distinctly binds β-tubulin at a site that differs from other antimicrotubule inhibitors, pausing microtubules for an extended amount of time and arresting them in mitosis, all without significant impact to the monomer/polymer equilibrium or total tubulin polymer mass within cells (168, 169). Compared to other microtubule inhibitors, Noscapine selective nature allows for less toxicity and no peripheral neuropathy, hypothesized to arise from dysfunctional cell cycle checkpoint mechanisms in tumor cells that make them more vulnerable to mitotic slippage and cell death upon exposure (168, 169).

Noscapine induces S-phase arrest and autophagic changes when inhibiting the growth of C6 GBM cells *in vitro (*168–170). Previous studies also noted its ability to sensitize GBM cells to taxane and radiation (168, 170). *In vivo* murine models, C6 GBM tumor volume was significantly reduced by 78% when given daily oral noscapine treatment (168, 169). No hepatic, splenic, hemopoietic, or duodenal toxicity was noted in this study (168, 169). Under hypoxic conditions, Noscapine was found to inhibit HIF-1α nuclear accumulation while targeting it for proteasomal degradation in human glioma cell lines U87MG and T98G (168, 171). Noscapine also acts as an indirect anti-angiogenic agent by decreasing transcription of HIF-1α and ultimately leading to reduced levels of VEGF (168, 171).

Additionally, Noscapine carries synergistic activity when used with conventional chemotherapies. A previous study analyzing U87MG human GBM cells *in vitro* found that when treated with a specific concentration of noscapine, the anti-tumor effects of TMZ, Bischloroethyl Nitrosourea (BCNU), and cisplatin were increased (168, 172). This effect was also observed in tumor xenografts treated with Noscapine combined with TMZ or Cisplatin, increasing apoptosis and decreasing proliferation (168, 172). Transcription factor NF-κB inducible and constitutive activity, crucial for GBM proliferation and radioresistance, is selectively blocked by noscapine (168). In GBM cell lines and pediatric glioma cells, newer analogs of noscapine (9-chloronoscapine and targetin) provide strongly improved tumoricidal effects and induced apoptosis (168). These analogs also provide the anti-inflammatory effects lacking in other antineoplastic agents (168). Further research regarding noscapine includes delivery methods like nano-liposomes and its human bitter taste receptor (Tas2R14) agonistic activity, which can induce tumor cell apoptosis (168).

Beyond noscapine, several other small-molecule inhibitors targeting GBM hypoxia and related pathways are under investigation, as outlined in **Table 1**.

**Table 1:** Small molecule inhibitors that target various pathways in GBM.

Pathway/target	Role in GBM	Inhibitors/agents	Mechanism/notes	References
SRC kinase	Mediates signaling pathways for migration, invasion, and survival	Pyrazolo[3,4-d]pyrimidines, Si306, Pro-Si306	Inhibit focal adhesion kinase, blocking invasion (*in vitro* & *in vivo*). Si306 may overcome multidrug resistance by inhibiting P-gp.	(173)
PI3K/Akt/mTOR	Central to GBM adaptation to hypoxia; often hyperactivated	Celastrol, Isolinderalactone, Metformin	Celastrol/isolinderalactone reduces angiogenesis & vasculogenic mimicry. Metformin suppresses pathway, reverses chemoresistance under hypoxia, increases sensitivity to TMZ.	(156)
TGF-β signaling	Drives mesenchymal shift and treatment resistance	Galunisertib, Disulfiram (in combination)	Galunisertib (anti-angiogenic, TGF-β receptor inhibitor). Disulfiram sensitizes treatment-resistant GBMs to TGF-β receptor inhibitors.	(174)
Integrins (αvβ3, αvβ5)	Mediate cell-cell and stromal interactions, invasion, survival	Cilengitide (C27H40N8O7)	Pentapeptide inhibitor of αvβ3/αvβ5. Restricts EGFRvIII/integrin β3 complex under hypoxia, leading to tumor regression, inhibites angiogenesis in xenografts.	(175)
Mitochondrial metabolism (antiparasitics)	Target tumor bioenergetics & oxygen consumption	Atovaquone, Doramectin, Ivermectin	Atovaquone: STAT3 inhibition, decreasing viability. Doramectin: regulates autophagy, decreasing tumor survival. Ivermectin: increases superoxide, induces oxidative stress, leading to mitochondrial dysfunction.	(156)

### 4.6 VEGF inhibitors reducing angiogenesis-driven hypoxia

GBM tumors tend to be poorly perfused as a result of their dysfunctional and abnormal vasculature, paradoxically contributing to an aggressive cycle of hypoxia it tries to alleviate with angiogenesis (157, 163). Bevacizumab (Bev) is a monoclonal antibody that targets GBM angiogenesis by blocking VEGF signaling pathways that ultimately decrease blood supply to the tumor (39, 156–158, 160). With significantly increased progression-free survival (PFS) rates and positive radiological responses, Bev was FDA-approved to be used as a second-line treatment or in combination with first-line treatments for recurrent GBM (156, 160, 176, 177).

When combining Bev with re-irradiation to treat recurrent GBM, She et al. have reported good patient tolerability, increased PFS, and improved OS (178). However, not many studies reflect similar outcomes to this. Paradoxically, Bev was also found to potentially cause a more hypoxic GBM TME that enhances invasion and resistance (179). Studies supporting this possible outcome reported post-Bev-treated animals having metabolic profiles (increased lactate, creatine, and choline) indicative of increased hypoxia (159, 179, 180). These findings underscore the paradox of anti-angiogenic therapy: while reducing tumor vasculature may slow growth initially, it can also select for more aggressive, metabolically adaptable tumor cells.

Considering the current literature, interpreting outcomes of Bev has been complicated by the variability in treatment protocols among various studies, to where definitive conclusions cannot be clearly drawn. Further research is needed to understand the relationship between factors like tumor vasculature, hypoxia, and response to antiangiogenesis therapy in GBM.

## 5 Future directions and conclusion

Despite decades of research, GBM remains one of the most challenging cancers to treat with notoriously poor clinical outcomes and low survival rates. As discussed in this review, increased recognition of the important role of metabolic reprogramming in the pathophysiology of GBM has highlighted new opportunities to exploit the tumor’s bioenergetics as potential treatment vulnerabilities. Growing evidence indicates that targeting these metabolic dependencies may offer therapeutic benefit, whether through dietary approaches like the ketogenic diet, pharmacologic inhibition of glycolysis and glutaminolysis, or disruption of mitochondrial activity and hypoxia/angiogenesis pathways. Future research is needed to investigate the value of integrating these interventions with the established regimens of surgery, radiation, and chemotherapy. However, it is important to note that clinical translation of these approaches does face several challenges: profound metabolic and molecular heterogeneity between patients (and even within tumors), difficulties in achieving adequate drug penetration across the blood-brain barrier, and the risk of toxicity in normal cerebral tissue. Several emerging research areas hold particular promise, such as tailored therapies based on molecularly defined subtypes of GBM (e.g., IDH-mutant vs. wild-type, EGFR-amplified vs. mesenchymal phenotypes) and using metabolic profiling for personalized treatment selection. In summary, while GBM’s unique metabolic characteristics confer a significant survival advantage to tumor cells, they also expose a key vulnerability. Ongoing efforts by researchers to further optimize metabolic targeting within a personalized treatment framework may have the potential to transform the therapeutic landscape of this otherwise devastating disease.
